# Unexpected Enhancement of HDACs Inhibition by MeS Substitution at C-2 Position of Fluoro Largazole

**DOI:** 10.3390/md18070344

**Published:** 2020-06-30

**Authors:** Bingbing Zhang, Zhu-Wei Ruan, Dongdong Luo, Yueyue Zhu, Tingbo Ding, Qiang Sui, Xinsheng Lei

**Affiliations:** 1 School of Pharmacy, Fudan University, 826 Zhangheng Road, Pudong Zone, Shanghai 201203, China; 14211030012@fudan.edu.cn (B.Z.); 13301030004@fudan.edu.cn (Z.-W.R.); 18211030012@fudan.edu.cn (Y.Z.); tbding@shmu.edu.cn (T.D.); 2 School of Medicine and Pharmacy, Ocean University of China, Qingdao 266003, China; luck_luodong@163.com; 3 China State Institute of Pharmaceutical Industry, No. 285 Gebaini Road, Pudong Zone, Shanghai 201203, China; chem_sq@163.com; 4 Key Laboratory of Synthetic Chemistry of Natural Substances, Shanghai Institute of Organic Chemistry, Chinese Academy of Sciences, Shanghai 200032, China

**Keywords:** marine natural product, largazole, HDAC inhibitors, modification, fluoro olefin

## Abstract

Given our previous finding that fluorination at the C18 position of largazole showed reasonably good tolerance towards inhibitory activity and selectivity of histone deacetylases (HDACs), further modification on the valine residue in the fluoro-largazole’s macrocyclic moiety with S-Me l-Cysteine or Glycine residue was performed. While the Glycine-modified fluoro analog showed poor activity, the S-Me l-Cysteine-modified analog emerged to be a very potent HDAC inhibitor. Unlike all previously reported C2-modified compounds in the largazole family (including our recent fluoro-largazole analogs) where replacement of the Val residue has failed to provide any potency improvement, the S-Me l-Cysteine-modified analog displayed significantly enhanced (five–nine-fold) inhibition of all the tested HDACs while maintaining the selectivity of HDAC1 over HDAC6, as compared to largazole thiol. A molecular modeling study provided rational explanation and structural evidence for the enhanced inhibitory activity. This new finding will aid the design of novel potent HDAC inhibitors.

## 1. Introduction

Both histone acetyltransferases (HATs) and histone deacetylases (HDACs) play a key role in the regulation of histone-tailed lysine acetylation status, which is closely associated with cell processes [1]. Up to now, 18 HDAC isoforms have been identified and divided into four classes, including the Zn^2+^-dependent Class I (HDAC1, 2, 3, 8), Class II (HDAC4–7, 9–10), Class IV (HDAC11), and NAD+- dependent Class III (namely SIRT1-7) [2]. Although the function of individual HDAC isoform is not fully understood in cells, HDACs have become an appealing target in cancer therapy through balancing histone hypoacetylation and overexpression of HDACs in multiple cancers. Currently, several Zn^2+^-dependent HDAC inhibitors, such as SAHA (Vorinostat), Belinostat, Panobinostat, Chidamide and Romidepsin (FK228), have been approved for the treatment of cutaneous T-cell lymphoma. However, these inhibitors are pan-selective inhibitors [3,4]. The current trend is to explore potent and selective HDAC inhibitors [5,6,7].

**Figure 1:**
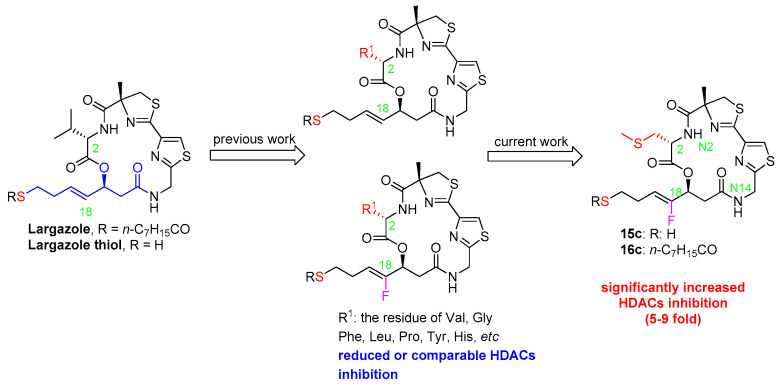
Largazole and its analogs modified at C-2 position.

Largazole, as a potent and selective Class I HDAC inhibitor, was discovered in 2008 by Leusch et al. [8,9]. Due to its superior anticancer properties, this marine natural product has attracted widespread attention in the medicinal chemistry community [10,11,12,13,14,15]. In order to improve the inhibitory activity and selectivity of HDACs, great efforts have been made to modify the macrocyclic moiety, the hydrophobic linker and the warhead of largazole. Thus far, almost all the modifications on the linker and the warhead cause obvious losses in activity against HDACs, possibly due to preventing the thiol group to efficiently penetrate into the narrow channel containing the Zn^2+^ ion of HDACs and to coordinate it with the optimal geometry. In contrast, the modifications on the macrocyclic moiety seem to be allowable to some extent. Among them, certain variations in valine residue (Val) at the C2 position of largazole may maintain the HDACs inhibition despite with a little sacrifice in its potency or isoenzyme selectivity (Figure 1). For example, Hong and Leusch’s group reported that replacement of Val with Ala decreased the activity by three-fold in growth inhibition of HCT-116 [16]. Subsequently, they introduced aromatic (Phe, Tyr), acidic (Asp) or basic (His) amino acid residues at that position, but all the analogs exhibited reduced activity in HDAC1 [17]. Based on the hypothesis of potentially hydrophobic interactions between Val and the surrounding residues of HDAC1 (Tyr196 and Leu263), Jiang et al. replaced Val with Leu, Phe and Tyr, reporting that Tyr had about five-fold higher GI_50_ values of HCT116 and A549 cells compared to largazole despite with an increasing selectivity over normal cells [18]. Ganesan’s and Williams’ groups reported that replacement with Gly or Pro resulted in a significantly decreased inhibition against HDACs, respectively [19,20]. Recently, Hong’s group investigated again the effect of the replacement of Val with Phe, Tyr, Asp or His on both the activity and selectivity of the class I HDACs, and the observed His substitution showed comparable activity, and a slight selectivity towards HDAC1 over HDAC2–3. This slight selectivity was hypothesized to be resulted from a possible hydrogen-bond ability of His. However, when they replaced Val with a set of residues tagged with the terminal amine or amides, the results suggested that the hydrogen bonding interaction did not play an essential role in HDAC inhibition [21]. All these works indicated that the structural optimization at the C2 position appeared not to improve the potency of HDACs inhibition. 

Recently, we discovered that the modification on the linker of largazole was allowable in some cases (Figure 1) [22,23,24]. For example, fixing fluorine at C18 position of the linker could keep almost the same inhibitory activity and selectivity of HDACs. Subsequently, the replacement of Val with Phe gave slightly reduced inhibition of HDACs, similar to the previous observations reported for largazole [23]. As our research continued, we discovered to our surprise that the replacement of Val with S-Me Cys significantly enhanced the inhibition of HDACs, whereas the replacement with Gly significantly decreased the activity. With an expanded research program under way based on this observation, we would like to communicate here our preliminary results and provide rational explanation for the unexpected results through a molecular modeling study. To the best of our knowledge, the S-Me Cys modification is the first reported example where replacement of the Val residue has led to significant enhancement in HDACs inhibition. We believe this insight may enable the design of highly potent HDAC inhibitors.

## 2. Results and Discussion

### 2.1. Chemistry

**Scheme 1:**
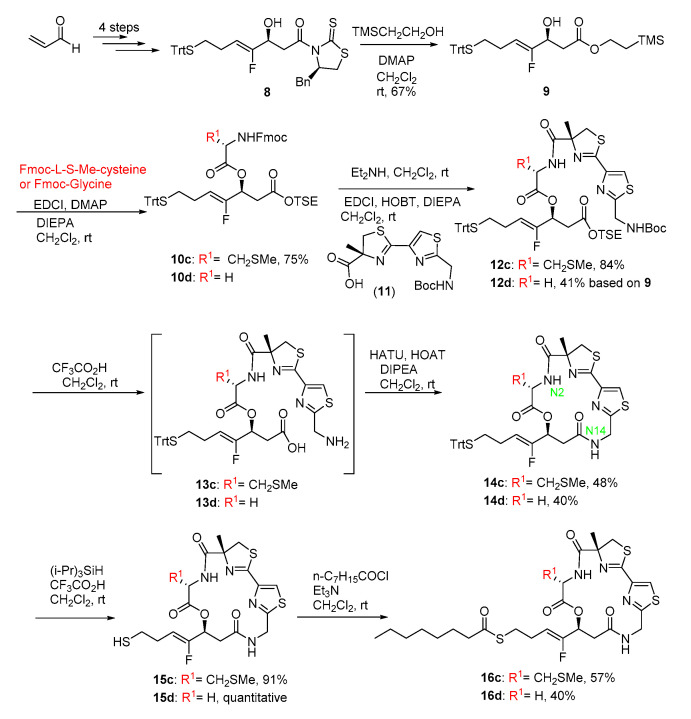
Synthesis of the analogs of Largazole. The red R^1^ is the different substituent and the green number is the atom code in the molecule.

In our previous paper [23], we synthesized the two fluoroolefin analogs of largazole with Val and Phe residue at C2 position. The key macrolactamization was chosen at N2 position, but the yields were poor (less than 31% yield). As an alternative, the key macrolactamization for the two new fluoroolefin analogs in this study was arranged at N14 position with an intend to improve the yield of the key step. The evolved synthetic route is depicted in Scheme 1. 

The key fluoro fragment (**8**) was prepared from acrolein via four steps according to our previous method [23]. Alcoholysis of **8** with TMSCH_2_CH_2_OH in the presence of DMAP provided **9** in good yield (67%). Condensation of **9** with S-Me Fmoc-l-Cystine or Fmoc-Glycine in the presence of EDCI, DMAP and DIEPA afforded ester **10c** and **10d** (yield: 75% for **10c**). Removal of Fmoc group of **10c** or **10d** with Et_2_NH in CH_2_Cl_2_ and subsequent condensation with the acid (**11**) resulted in the linear precursors (**12c**, 84%; **12d**, 41% based on **9**). Deprotection of Boc group of **10c** or **10d** with CF_3_CO_2_H in CH_2_Cl_2_ and subsequent release of free acid provided the linear depsipeptides (**13c** and **13d**) exposed at the N- and C-terminus, respectively. The depsipeptides were then subjected to the optimal cyclization condition (HATU/HOAT/DIPEA in anhydrous CH_2_Cl_2_ solution with a diluted concentration of about 0.001 M). The macrolactamization yields were 48% for **14c** and 40% for **14d** in two steps, respectively. Compared to our previous cyclization at N2 position, cyclizing at N14 position afforded apparently improved yield. Following our previously reported procedure [24], the free thiol **15c** and **15d** were obtained through deprotection of Trt group from **14c** and **14d**, respectively. The subsequent acylation with *n*-C_7_H_15_COCl under the standard condition led to the final fluoro analogs **16c** and **16d** (yields: 57%, 40%), respectively.

### 2.2. Biology

It is well-known that largazole is a prodrug species for a beneficial cell permeability and its free thiol is indeed the activated species for the inhibition against HDACs [10]. Given the instability of the free thiol for storage, the in vitro cell assays were used as the initial screening by our well-established cell tests (A549, HCT116, MDA-MB-231 and SK-OV-3 cells), aiming at speedy identification of potent HDAC inhibitors. The preliminary results were shown in Table 1, using largazole as the positive control compound.

Compared with largazole, **16c** displayed the obvious growth inhibition towards A549, HCT116 and SK-OV-3 cells with IC_50_ values ranged with 1.41–3.13 μM, but not towards MDA-MB-231 (IC_50_: >10µM). In contrast, **16d** almost lost the growth inhibition towards most of those cells, and only maintained a slight growth inhibition towards HCT116 (IC_50_: 6.62 µM), indicating that **16d** was a less potent HDAC inhibitor, which was consistent with previous reports [19,20], where both Ganesan’s and Williams’ groups demonstrated that the replacement of Val with Gly in the case of largazole resulted in a significantly decrease of inhibition against HDACs.

To further confirm the growth inhibition of **16c** in cellular assays, we tested **16c** and largazole with other cells. As shown in Table 2, **16c** displayed growth inhibition against Hela, Eca-109, Bel 7402 and U937 cells with high potencies that are quite identical to largazole.

Based on the obvious growth inhibition of **16c** in in vitro cell assays, we next performed in vitro enzyme assays with the correspond active species **15c**, and the data were compared with those of our previous compounds. The results are shown in Table 3.

In sharp contrast to the cellular assays where **16c** exhibited identical or slightly inferior activities as compared to largazole, in the enzymatic assays, compound **15c** surprisingly displayed significantly increased inhibition towards HDACs, with the IC_50_ values of HDAC 1, 2, 3, 8 and 6 being 0.27, 1.33, 2.33, 0.44 and 23.91 nM, respectively. It was five–nine-fold more potent towards all the tested HDACs when compared with largazole thiol. Moreover, the selectivity of HDAC1 over HDAC6 remained unchanged (selectivity: 89). Notably, we demonstrated earlier that the fluoro analogs (**15a** and **15b**) exhibited slightly less activities than largazole thiol, which was consistent with all previous observations made with substitutions at C2 for largazole [16,17,18,19,20,21]. We wanted to compare **15c** with similar largazole compounds. However, a literature search revealed no prior report on modification at C2 position with a sulphur-containing substituent. **15c** appeared to be the first example of such compounds in the largazole family. The surprisingly enhanced activity of **15c** might be attributed to the variation in Val at the C2 position, suggesting that the MeS group of **15c** played an important role in the interaction between **15c** and HDACs. This was confirmed subsequently by the results from a molecular modeling study.

### 2.3. Molecular Modeling Study

To gain some structural insight on the increased inhibitory effects of **15c** on the tested HDACs, molecular docking was performed by using MOE 2019 with MMF94 force field. The crystal structures of HDACs were downloaded from the Protein DataBank (PDB, http://www.rcsb.org), and were used to investigate the binding modes of **15c** with HDAC1, HDAC6 and HDAC8, respectively (PDB code: HDAC1, 5ICN; HDAC6, 5EDU; HDAC8, 4RN0). 

**Figure 2:**
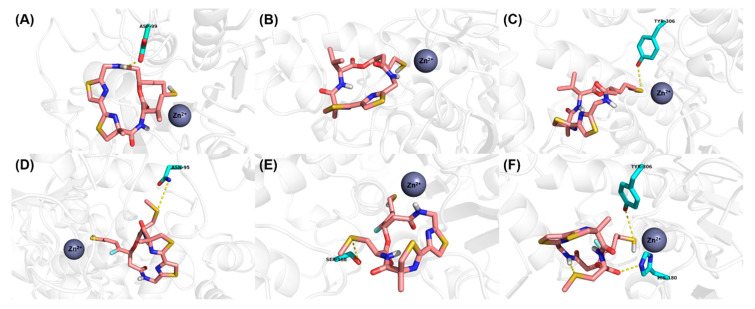
Proposed binding mode of Largazole thiol (carmine carbon sticks) with (**A**) HDAC1 (5ICN), (**B**) HDAC6 (5EDU) and (**C**) HDAC8 (4RN0). Proposed binding mode of **15c** (carmine carbon sticks) with (**D**) HDAC1 (5ICN), (**E**) HDAC6 (5EDU) and (**F**) HDAC8 (4RN0). Atom color code: red = oxygen, blue = nitrogen, white = hydrogen, yellow = sulfur, cyan = fluorine. Hydrogen bonds between the ligand and receptor are indicated by yellow dashed lines.

The binding modes of largazole thiol in HDAC1, HDAC6 and HDAC8 indicated that the thiol side chain could coordinate to the catalytic Zn^2+^ ion and the overall metal coordination geometry was nearly perfectly tetrahedral [25]. Addtionally, largazole thiol formed hydrogen bond interactions with ASP99 in HDAC1 and TYR306 in HDAC8, respectively (Figure 2A–C). For **15c**, which was derived from largazole thiol, additional hydrogen bonds were formed between sulphur atom in the methylthio group and ASN95 in HDAC1 and SER568 in HDAC6, respectively (Figure 2D,E). Interestingly, the introduction of MeS substitution resulted in orientation change of the ester bond at C1 position, and then made an oxygen atom of the carboxyl group form a new hydrogen bond with HIS180 of HDAC8 (Figure 2F). These additional interactions are likely the factors that have led to significant improvement in **15c**’s potency towards HDACs. These findings from molecular modeling study confirmed our hypothesis about the key role that MeS group played, and provided telling structural evidence for the significantly increased inhibition of **15c** towards HDACs.

## 3. Materials and Methods 

### 3.1. Chemistry

The chemicals and reagents were purchased from Acros (Shanghai, China), Alfa Aesar (Shanghai, China), and National Chemical Reagent Group Co. Ltd., P. R. China (Shanghai, China), and used without further purification. Anhydrous solvents (THF, MeOH, DMF, CH_2_Cl_2_ and CH_3_CN) used in the reactions were dried and freshly distilled before use. All the reactions were carried out under Ar atmosphere, otherwise stated else. The progress of the reactions was monitored by TLC (silica-coated glass plates) and visualized under UV light, and by using iodine or phosphomolybdic acid. Melting points were measured on an SGW X-4 microscopy melting point apparatus without correction. ^1^H NMR and ^13^C NMR spectra were recorded either on a 400 MHz Varian Instrument at 25 °C or 600 MHz Bruker Instrument at 25 °C, using TMS as an internal standard, respectively. Multiplicity is tabulated as s for singlet, d for doublet, dd for doublet of doublet, t for triplet, and m for multiplet. The original spectra of the relative compounds could be found in Supplementary Materials. HRMS spectra were recorded on Finnigan-Mat-95 mass spectrometer, equipped with ESI source. Largazole, its free thiol and key intermediates (compound **8** and **11**) were prepared according to our previous method [22,23,24]. 

#### 3.1.1. 2-(Trimethylsilyl)ethyl (S,Z)-4-fluoro-3-hydroxy-7-(tritylthio)hept-4-enoate (**9**)

To a solution of **8** (0.595 g, 0.95 mmoL, 1.0 equiv.) and DMAP (0.183 g, 1.5 mmoL, 1.5 equiv.) in CH_2_Cl_2_ (DCM, 20 mL), Me_3_CH_2_CH_2_OH was added (1.36 mL, 9.5 mmol, 10 equiv.), and the resulting solution was stirred at room temperature (rt) overnight. Concentration in vacuo left the residue, which was purified by column chromatography, giving compound **9** (0.341 mg) with a 67% yield as colorless oil. *R*_f_ = 0.17 [petroleum ether (PE)/ethyl acetate (EA) 10/1]. ^1^H-NMR (600 MHz, CDCl_3_): δ 7.50–7.13 (m, 15H, Ar*H*), 4.81 (dt, *J* = 37.2, 7.2 Hz, 1H, C*H*=CF), 4.46 (m, 1H, C*H*OH), 4.20 (m, 2H, OC*H*_2_CH_2_TMS), 3.14 (brs, 1H, O*H*), 2.63 (dd, *J* = 16.2, 3.6 Hz, 1H, C*H*_2_CO), 2.58 (dd, *J* = 16.2, 9.0 Hz, 1H, C*H*_2_CO), 2.20 (t, *J* = 7.2 Hz, 2H, SC*H*_2_CH_2_), 2.14 (t, *J* = 7.2 Hz, 2H, SCH_2_C*H*_2_), 0.99 (t, *J* = 8.4 Hz, 2H, OCH_2_C*H*_2_TMS), 0.03 (s, 9H, Si*Me*_3_). ^13^C-NMR (150 MHz, CDCl_3_): δ 171.4, 157.9 (d, *J* = 255 Hz), 144.2, 128.9, 127.2, 126.0, 104.2, 66.1 (d, *J* = 27 Hz), 62.7, 37.9, 30.8, 22.0, 16.6, 2.1. ^19^F NMR (376 MHz, CDCl_3_): δ −123.24 (dd, *J* = 37.6, 11.2 Hz). ESI (*m/z*): [M + H]^+^ 537.4.

#### 3.1.2. 2-(Trimethylsilyl)ethyl (S,Z)-3-((N-(((9H-fluoren-9-yl)methoxy)carbonyl)-S-methyl-l-cysteinyl)oxy)-4-fluoro-7-(tritylthio)hept-4-enoate (**10c**)

To a solution of **9** (0.690 g, 1.28 mmol, 1.0 equiv.) in DCM (10 mL) a solution of S-Me Fmoc-l-Cysteine was added (2.38 g, 6.42 mmol, 5.0 equiv.); DMAP (0.016 g, 0.128 mmol, 0.1 equiv.) and EDCI (1.48 g, 7.7 mmol, 6.0 equiv.) was added in DCM (20 mL) at rt, and DIPEA (1.30 mL, 7.7 mmol, 2.5 equiv.) was added to the stirring solution. The reaction was stirred for 18 h. This solution was quenched with a NH_4_Cl aqueous solution and separated. The aqueous phase was extracted with DCM (30 mL × 3) and the combined organic phase was washed with H_2_O, brine, dried with Na_2_SO_4_ and filtered. After the removal of the solvent, the resulting crude was purified by flash chromatography on silica gel, eluting with PE/EA (5/1) to afford **10c** (0.856 g, 75%) as a white foamy solid. *R*_f_ = 0.20 (PE/EA 5/1). ^1^H-NMR (600 MHz, CDCl_3_): δ 7.80–7.10 (m, 23H, Ar*H*), 6.64 (m, 1H, C*H*OC=O), 5.37 (d, *J* = 8.4 Hz, 1H, N*H*), 4.88 (dt, *J* = 36, 7.2 Hz, 1H, C*H*=CF), 4.49 (m, 1H, C*H*CH_2_SMe), 4.40 (m, 2H, OC*H*_2_CH[Fmoc]), 4.21 (m, 1H, OCH_2_C*H*[Fmoc]), 4.16 (m, 2H, OC*H*_2_CH_2_TMS), 2.80 (dd, *J* = 16.8, 8.4 Hz, 1H, C*H*_2_CO), 2.68 (dd, *J* = 16.8, 4.8 Hz, 1H, C*H*_2_CO), 2.42 (m, 1H, CHC*H*_2_SMe), 2.20–2.14 (m, 4H, SCH_2_C*H*_2_), 2.03 (s, 3H, CHCH_2_S*Me*), 1.91 (m, CHC*H*_2_SMe), 0.96 (t, *J* = 8.4 Hz, 2H, OCH_2_C*H*_2_TMS), 0.01 (s, 9H, Si*Me*_3_). ^13^C-NMR (150 MHz, CDCl_3_): δ 170.2, 168.4, 155.1, 153.9 (d, *J* = 257 Hz), 144.1, 143.2, 143.0, 140.7, 128.9, 127.2, 127.1, 126.4, 126.0, 124.4, 119.3, 108.8 (d, *J* = 14 Hz), 68.8 (d, *J* = 29 Hz), 66.4, 66.2, 62.8, 52.4, 46.5, 35.6, 31.2, 30.4, 22.2, 16.6, 14.7, −2.1. ^19^F NMR (376 MHz, CDCl_3_): δ −123.28 (dd, *J* = 37.6, 18.8 Hz). HRMS-ESI (*m/z*): [M + H]^+^ calcd. for C_50_H_54_FNO_6_S_2_SiH: 876.3219, found: 876.3223.

#### 3.1.3. 2-(Trimethylsilyl)ethyl (S,Z)-3-((N-((R)-2′-(((tert-butoxycarbonyl)amino)methyl)-4-methyl-4,5-dihydro-[2,4′-bithiazole]-4-carbonyl)-S-methyl-L-cysteinyl)oxy)-4-fluoro-7-(tritylthio)hept-4-enoate (**12c**)

To a solution of **10c** (0.890 g, 0.90 mmol, 1.0 equiv.) in DCM (60 mL) was added Et_2_NH (4.74 mL, 29 mmol, 10 equiv.) was added at rt. After stirring for 2 h and removal of the solvent, the residue was purified by flash chromatography on silica gel, eluting with PE/EA (3/2) to afford the corresponding amine (0.465 g, 77%) as a white foamy solid. 

A mixture of compound **11** (0.196 g, 0.55 mmol, 1.0 equiv.), the amine (0.438 g, 0.66 mmol, 1.2 equiv.), HOBT (0.089 g, 0.66 mmol, 1.2 equiv.) and EDCI (0.126 g, 0.66 mmol, 1.2 equiv.) was dissolved in dry DCM (50 ml) and stirred at rt for 3 h. The solution was concentrated under reduced pressure, and diluted with EA (150 mL), washed saturated NaHCO_3_ aqueous solution and brine successively, dried over Na_2_SO_4_ and filtered. After removal of the solvent, the residue was purified by flash chromatography on silica gel, eluting with PE/EA (5/2) to afford **12c** (84% based on **10c**) as a white foamy solid. *R*_f_ = 0.30 (PE/EA 5/2). ^1^H-NMR (600 MHz, CDCl_3_): δ 7.87 (s, 1H, Ar*H*), 7.5–7.10 (m, 17H, Ar*H*, 2N*H*), 5.60 (ddd, *J* = 19.8, 8.4, 5.4 Hz, 1H, C*H*OC=O), 5.29 (dd, *J* = 36, 7.2 Hz, 1H, C*H*=CF), 4.68 (m,1H, C*H*CH_2_SMe), 4.62 (d, *J* = 5.4 Hz, 2H, C*H*_2_NHCOCMe_3_), 4.18 (m, 2H, OC*H*_2_CH_2_SiMe_3_), 4.04 (d, *J* = 11.4 Hz, 1H, C_q_C*H*_2_S), 3.32 (d, *J* = 11.4 Hz, 1H, C_q_C*H*_2_S), 2.81 (dd, *J* = 16, 8.4 Hz, 1H, C*H*_2_CO_2_), 2.70 (dd, *J* = 16, 8.4 Hz, 1H, C*H*_2_CO_2_), 2.39 (m, 2H, CHC*H*_2_SMe), 2.20–2.00 (m, 4H, Ph_3_CSC*H*_2_C*H*_2_), 1.96 (s, 3H, CHCH_2_S*Me*), 1.57 (s, 3H, C_q_C*H*_3_), 1.46 (s, 9H, CH_2_NHCOC*Me*_3_), 0.97 (t, *J* = 8.4 Hz, 3H, OCH_2_C*H*_2_SiMe_3_), 0.03 (s, 9H, OCH_2_CH_2_Si*Me*_3_). ^13^C-NMR (150 MHz, CHCl_3_): δ 173.6, 168.9, 168.7, 167.8, 164.5, 155.2 (d, *^1^J*_C-F_ = 256 Hz), 147.4, 129.5, 127.9, 126.6, 124.3, 109.2 (d, *^2^J*_C-F_ = 12.4 Hz), 84.3, 69.9 (d, *^3^J*_C-F_ = 29.6 Hz), 57.5, 43.3, 41.1, 38.6, 37.4, 34.2, 31.1, 29.7, 24.1, 22.8, 18.8, 16.5, 14.2. ^19^F NMR (376 MHz, CDCl_3_): δ -124.91 (dd, *J* = 37.6, 18.8 Hz). ESI (*m/z*): [M + Na]^+^ 1015.4.

#### 3.1.4. 2-(Trimethylsilyl)ethyl (S,Z)-3-((((R)-2′-(((tert-butoxycarbonyl)amino)methyl)-4-methyl-4,5-dihydro-[2,4′-bithiazole]-4-carbonyl)glycyl)oxy)-4-fluoro-7-(tritylthio)hept-4-enoate (**12d**)

Compound **12d** was prepared from Fmoc-Glycine in 41% yield based on **9** using the same procedure for **12c**. ^1^H NMR (600 MHz, CDCl_3_) δ 7.85 (s, 1H, Ar*H*), 7.53–7.09 (m, 16H, Ar*H*, N*H*), 5.68 (ddd, *J* = 19.7, 8.8, 5.2 Hz, 1H, C*H*OC=O), 5.36 (brs, 1H, N*H*), 4.88 (dt, *J* = 36.0, 8.0 Hz, 1H, C*H*=CF), 4.61 (d, *J* = 6.3 Hz, 2H, C*H*_2_NHCOCMe_3_), 4.25–4.08 (m, 2H, OC*H*_2_CH_2_SiMe_3_), 4.12–3.94 (m, 2H, NHC*H*_2_CO_2_), 3.74 (d, *J* = 11.7 Hz, 1H, C_q_C*H*_2_S), 3.32 (d, *J* = 11.5 Hz, 1H, C_q_C*H*_2_S), 2.77 (dd, *J* = 16.6, 8.7 Hz, 1H, C*H*_2_CO_2_), 2.66 (dd, *J* = 16.5, 5.0 Hz, 1H, C*H*_2_CO_2_), 2.12 (dd, *J* = 25.5, 7.1 Hz, 4H, Ph_3_CSC*H*_2_C*H*_2_), 1.58 (s, 3H, C_q_C*H*_3_), 1.46 (s, 9H, CH_2_NHCOC*Me*_3_), 1.04–0.88 (m, 2H, OCH_2_C*H*_2_SiMe_3_), 0.02 (s, 9H, OCH_2_CH_2_Si*Me*_3_). ^13^C NMR (150 MHz, CDCl_3_) δ 174.7, 169.7, 169.1, 168.2, 163.3, 155.6, 154.5 (d, *J* = 258.2 Hz), 148.5, 144.7, 129.5, 127.8, 126.6, 121.9, 108.9 (d, *J* = 13.7 Hz), 84.9, 80.4, 69.1 (d, *J* = 30.2 Hz), 66.6, 63.4, 42.3, 41.2, 41.0, 36.3, 31.0, 28.3, 24.7, 22.8, 17.2, -1.5. ^19^F NMR (376 MHz, CDCl_3_): 124.96 (m). ESI (*m/z*): [M + Na]^+^ 955.5.

#### 3.1.5. (1^2^Z,2^2^Z,2^4^R,5R,8S)-8-((Z)-1-fluoro-4-(tritylthio)but-1-en-1-yl)-2^4^-methyl-5-((methylthio)methyl)-2^4^,2^5^-dihydro-7-oxa-4,11-diaza-1(4,2),2(2,4)-dithiazolacyclododecaphane-3,6,10-trione (**14c**)

**12c** (0.446 g, 0.44 mmol, 1.0 equiv.) was dissolved in a solution of CF_3_CO_2_H (5 mL) and dry DCM (20 mL), and stirred at rt overnight. After removal of the solvent under reduced pressure, and the crude amino acid (about 0.408 g) was directly used for the next step.

The amine acid was dissolved in anhydrous DMF (40 mL) and then DIEPA (0.50 mL, 2.66 mmol, 6.0 equiv.) was added to the solution at rt in order to prepare the amine solution. Next, the amine solution was added dropwise to a stirring solution of HATU (0.337 g, 0.89 mmol, 2.0 equiv.) and HOBT (0.120 g, 0.89 mmol, 2.0 equiv.) in anhydrous DMF (80 mL) at rt over 3 h. The resulting solution continued to stir at rt overnight. The mixture was diluted with water (150 mL), and extracted with MeOBu^t^ (80 mL). The combined organic phase was washed with a saturated NaHCO_3_ aqueous solution (50 mL × 3), water (50 mL) and brine (50 mL). After drying by NaSO_4_, filtration and evaporation, the residue was purified by flash chromatography on silica gel, eluting with PE/EA (1/1) to afford **14c** (48% in 2 steps) as a white foamy solid. *R*_f_ = 0.20 (PE/EA 1/1). ^1^H-NMR (600 MHz, CDCl_3_): δ 7.75 (s, 1H, Ar*H*), 7.5–7.10 (m, 16H, Ar*H*, N*H*), 6.39 (m, 1H, N*H*), 5.70 (m, 1H, C*H*OC=O), 5.52 (dd, *J* = 17.4, 9.6 Hz, 1H, ArC*H*_2_NH), 4.96 (dd, *J* = 36, 7.2 Hz, 1H, C*H*=CF), 4.68 (dd, *J* = 12, 4.2 Hz, 1H, C*H*CH_2_SMe), 4.22 (dd, *J* = 17.4, 3.0 Hz, 1H, ArC*H*_2_NH), 4.08 (d, *J* = 11.4 Hz, 1H, C_q_C*H*_2_S), 3.20 (d, *J* = 11.4 Hz, 1H, C_q_C*H*_2_S), 3.13 (dd, *J* = 16, 11.4 Hz, 1H, C*H*_2_CO_2_), 2.70 (d, *J* = 16, 1H, C*H*_2_CO_2_), 2.30–1.90 (m, 6H, Ph_3_CSC*H*_2_C*H*_2_, CHC*H*_2_SMe), 1.96 (s, 3H, CHCH_2_S*Me*), 1.70 (s, 3H, C_q_C*H*_3_). ^13^C-NMR (150 MHz, CHCl_3_): δ 172.8, 168.3, 168.2, 167.2, 163.6, 154.1 (d, *^1^J*_C-F_ = 256 Hz), 146.8, 144.1, 128.9, 127.2, 126.0, 123.8, 108.8 (d, *^2^J*_C-F_ = 12 Hz), 83.6, 69.4 (d, *^3^J*_C-F_ = 30 Hz), 66.0, 51.8, 42.2, 40.6, 36.5, 30.9, 30.4, 27.8, 23.6, 22.2, 14.2. ^19^F NMR (376 MHz, CDCl_3_): δ −125.10 (dd, *J* = 37.6, 18.8 Hz). HRMS-ESI (*m/z*): [M + H]^+^ calcd. for C_39_H_39_FN_4_O_4_S_4_H: 775.1911, found: 775.1918.

#### 3.1.6. (1^2^Z,2^2^Z,2^4^R,8S)-8-((Z)-1-fluoro-4-(tritylthio)but-1-en-1-yl)-2^4^-methyl-2^4^,2^5^-dihydro-7-oxa-4,11-diaza-1(4,2),2(2,4)-dithiazolacyclododecaphane-3,6,10-trione (**14d**)

Compound **12d** was prepared in 40% yield using the same procedure for **12c**. ^1^H NMR (600 MHz, CDCl_3_) δ 7.72 (s, 1H, Ar*H*), 7.44–7.17 (m, 15H, Ar*H*), 7.03 (brs, 1H, N*H*), 6.55–6.46 (m, 1H, N*H*), 5.76 (dd, *J* = 22.6, 11.1 Hz, 1H, C*H*OC=O), 5.19 (dd, *J* = 17.4, 8.9 Hz, 1H, ArC*H*_2_NH), 4.94 (dt, *J* = 36.0, 8.0 Hz, 1H, C*H*=CF), 4.24 (dd, *J* = 17.4, 4.0 Hz, 1H, ArC*H*_2_NH), 4.13 (m, 1H, C*H*_2_CO_2_), 4.12 (d, *J* = 12.0 Hz, 1H, C_q_C*H*_2_S), 3.88 (dd, *J* = 18.7, 4.0 Hz, 1H, C*H*_2_CO_2_), 3.22 (d, *J* = 12.0 Hz, 1H, C*H*_2_CO_2_), 3.13 (dd, *J* = 16.9, 11.3 Hz, 1H, C*H*_2_CONH), 2.70 (d, *J* = 16.8 Hz, 1H, C*H*_2_CONH), 2.16–2.14 (m, 4H, Ph_3_CSC*H*_2_C*H*_2_), 1.82 (s, 3H, C_q_C*H*_3_). ^13^C NMR (150 MHz, CDCl_3_) δ 173.8, 169.0, 167.5, 165.9, 163.7, 154.7 (d, *J* = 258.2 Hz), 147.4, 144.7, 129.5, 127.8, 126.6, 124.6, 109.6 (t, *J* = 12.1 Hz), 84.3, 70.0 (d, *J* = 28.7 Hz), 66.6, 43.6, 42.0, 41.4, 37.1, 31.0, 25.2, 22.8. ^19^F NMR (376 MHz, CDCl_3_): δ −129.82 (dd, *J* = 37.6, 22.5 Hz). HRMS-ESI (*m/z*): [M + Na]^+^ calcd. for C_37_H_35_FN_4_O_4_S_3_Na: 737.1697, found: 737.1675. 

#### 3.1.7. (1^2^Z,2^2^Z,2^4^R,8S)-8-((Z)-1-fluoro-4-mercaptobut-1-en-1-yl)-2^4^-methyl-5-((methylthio)methyl)-2^4^,2^5^-dihydro-7-oxa-4,11-diaza-1(4,2),2(2,4)-dithiazolacyclododecaphane-3,6,10-trione (**15c**)

**14c** (0.050 mg, 0.63 mmol, 1.0 equiv.) was dissolved in dry DCM (10 mL) and cooled to 0 °C. The mixture was successively treated with *i*-Pr_3_SiH (26 µL, 0.13 mmol, 2.0 equiv.) and TFA (0.30 mL, 4.0 mmol, 6.7 equiv.). The reaction mixture was allowed to warm to rt and stirred for 1.5 h. The reaction was quenched with a saturated NaHCO_3_ solution (10 mL) and separated. The aqueous phase was extracted with DCM (10 mL × 3), and the combined layers was washed with brine, dried over Na_2_SO_4_ and filtered. After removal of the solvent, the residue was purified by flash chromatography on silica gel, eluting with DCM/MeOH (60/1) to afford **15c** in 91% yield as a white foamy solid. *R*_f_ = 0.30 (PE/EA 2/3). ^1^H-NMR (400 MHz, CDCl_3_): δ ^1^H-NMR (600 MHz, CDCl_3_): δ 7.78 (s, 1H, Ar*H*), 7.26 (d, *J* = 7.6 Hz, 1H, N*H*), 6.44 (dd, *J* = 10, 3.6 Hz, 1H, N*H*), 5.77 (ddd, *J* = 21.2, 11.2, 2.0 Hz, 1H, C*H*OC=O), 5.28 (dd, *J* = 17.6, 9.6 Hz, 1H, ArC*H*_2_NH), 5.11 (dt, *J* = 36, 7.6 Hz, 1H, C*H*=CF), 4.70 (m, 1H, C*H*CH_2_SMe), 4.27 (dd, *J* = 17.6, 3.6 Hz, 1H, ArC*H*_2_NH), 4.08 (d, *J* = 11.2 Hz, 1H, C_q_C*H*_2_S), 3.25 (d, *J* = 11.2 Hz, 1H, C_q_C*H*_2_S), 3.19 (dd, *J* = 16.8, 11.2 Hz, 1H, C*H*_2_CONH), 2.76 (dd, *J* = 16.8, 2.4 Hz, 1H, C*H*_2_CONH), 2.41 (m, 2H, C*H*_2_CH=CF), 2.30–1.90 (m, 4H, C*H*_2_SH, CHC*H*_2_SMe), 1.87 (s, 3H, CHCH_2_S*Me*), 1.85 (s, 3H, C_q_C*H*_3_). ^13^C-NMR (150 MHz, CHCl_3_): δ 174.1, 169.5, 169.4, 164.8, 155.9 (d, *^1^J*_C-F_ = 256 Hz), 147.9, 125.0, 109.3 (d, *^2^J*_C-F_ = 12 Hz), 84.8, 70.9 (d, *^3^J*_C-F_ = 30 Hz), 53.0, 43.4, 41.8, 37.8, 28.4, 24.8, 24.3, 15.6. ^19^F NMR (376 MHz, CDCl_3_): δ −124.79 (dd, *J* = 37.6, 18 Hz). HRMS-ESI (*m/z*): [M + H]^+^ calcd. for C_20_H_25_FN_4_O_4_S_4_H: 533.0815, found: 533.0819.

#### 3.1.8. S-((Z)-4-fluoro-4-((1^2^Z,2^2^Z,2^4^R,5R,8S)-2^4^-methyl-5-((methylthio)methyl)-3,6,10-trioxo-2^4^,2^5^-dihydro-7-oxa-4,11-diaza-1(4,2),2(2,4)-dithiazolacyclododecaphane-8-yl)but-3-en-1-yl) octanethioate (**16c**)

The free thiol **15c** (32 mg, 0.05mmol, equiv.) was dissolved in dry DCM (10 mL) and cooled to 0 °C. The mixture was successively treated with pyridine (23 µL, 0.29 mmol, 5.0 equiv.) and octanoyl chloride (29 µL, 0.17 mmol, 3.0 equiv.). The reaction was allowed to warm to rt and stirred for 2 h, and then quenched with a saturated NaHCO_3_ solution (10 mL) and separated. The aqueous phase was extracted with DCM (10 mL × 3), and the combined layers was washed with brine, dried over Na_2_SO_4_ and filtered. After removal of the solvent, the residue was purified by flash chromatography on silica gel, eluting with DCM/EA (1/1) to afford **16c** (57%) as a white foamy solid. *R*_f_ = 0.42 (DCM/EA 3/1). ${\left[\alpha \right]}_{20}^{D}$ 26.4 (c 0.34, CHCl_3_). ^1^H-NMR (600 MHz, CDCl_3_): δ 7.78 (s, 1H, Ar*H*), 7.24 (d, *J* = 11.4 Hz, 1H, N*H*), 6.54 (dd, *J* = 9.6, 3.6 Hz, 1H, N*H*), 5.76 (ddd, *J* = 21, 11.4, 2.4 Hz, 1H, C*H*OC=O), 5.30 (dd, *J* = 17.4, 9.6 Hz, 1H, ArC*H*_2_NH), 5.09 (dt, *J* = 36, 7.2 Hz, 1H, C*H*=CF), 4.71 (m, 1H, C*H*CH_2_SMe), 4.27 (dd, *J* = 17.5, 3.0 Hz, 1H, ArC*H*_2_NH), 4.10 (d, *J* = 11.4 Hz, 1H, C_q_C*H*_2_S), 3.28 (d, *J* = 11.4 Hz, 1H, C_q_C*H*_2_S), 3.19 (dd, *J* = 16.8, 11.4 Hz, 1H, C*H*_2_CONH), 2.90 (t, *J* = 7.2 Hz, 2H, SC*H*_2_CH_2_), 2.75 (dd, *J* = 16.8, 2.4 Hz, 1H, C*H*_2_CONH), 2.54 (t, *J* = 7.5 Hz, 2H, C*H*_2_COS), 2.40–2.30 (m, 2H, SCH_2_C*H*_2_), 2.30–1.90 (m, 2H, CHC*H*_2_SMe), 1.87 (s, 3H, CHCH_2_S*Me*), 1.86 (s, 3H, C_q_C*H*_3_), 1.70–1.6 (m, 2H, C*H*_2_), 1.40–1.10 (m, 8H, C*H*_2_), 0.89 (t, *J* = 6.0 Hz, 3H, C*H*_3_CH_2_). ^13^C-NMR (150 MHz, CDCl_3_): δ 198.6, 172.9, 168.3, 168.2, 167.3, 163.8, 154.7 (d, *J* = 256 Hz), 146.7, 123.9, 108.1 (d, *J* = 12 Hz), 83.6, 69. 5 (d, *J* = 30 Hz), 51.8, 43.5, 42.2, 40.6, 36.5, 30.9, 28.4, 27.2, 24.9, 24.1, 23. 6, 23.2, 21.9, 14.4, 13.4. ^19^F NMR (376 MHz, CDCl_3_): δ −124.69 (dd, *J* = 35.9, 20.9 Hz). HRMS-ESI (*m/z*): [M + Na]^+^ calcd. for C_28_H_39_FN_4_O_5_S_4_Na: 681.1680, found: 681.1693.

#### 3.1.9. S-((Z)-4-fluoro-4-((1^2^Z,2^2^Z,2^4^R,8S)-2^4^-methyl-3,6,10-trioxo-2^4^,2^5^-dihydro-7-oxa-4,11-diaza-1(4,2),2(2,4)-dithiazolacyclododecaphane-8-yl)but-3-en-1-yl) octanethioate (**16d**)

Compound **16d** was prepared in 40% yield from **14d** using the same procedure for **16c**. ${\left[\alpha \right]}_{20}^{D}\;$ 37.9 (c 0.14, CHCl_3_). ^1^H NMR (600 MHz, CDCl_3_) δ 7.71 (s, 1H, Ar*H*), 7.00 (m, 1H, N*H*), 6.60 (dd, *J* = 9.1, 4.1 Hz, 1H, N*H*), 5.78 (dd, *J* = 22.3, 11.1 Hz, 1H, C*H*OC=O), 5.18 (dd, *J* = 17.4, 8.8 Hz, 1H, ArC*H*_2_NH), 5.04 (dt, *J* = 36.1, 7.5 Hz, 1H, C*H*=CF), 4.24 (dd, *J* = 17.5, 4.0 Hz, 1H, ArC*H*_2_NH), 4.15 (m, 1H, C*H*_2_CO_2_), 4.10 (d, *J* = 11.7 Hz, 1H, C_q_C*H*_2_S), 3.87 (dd, *J* = 18.9, 3.1 Hz, 1H), 3.20 (d, *J* = 11.2 Hz, 1H, C_q_C*H*_2_S), 3.14 (dd, *J* = 16.0, 12.0 Hz, 1H, C*H*_2_CONH), 2.86 (t, *J* = 7.1 Hz, 2H, SC*H*_2_CH_2_), 2.71 (d, *J* = 16.9 Hz, 1H, C*H*_2_CO), 2.50 (t, *J* = 7.6 Hz, 2H, C_6_H_13_C*H*_2_CO), 2.41–2.27 (m, 2H, SCH_2_C*H*_2_), 2.11–1.87 (m, 2H), 1.79 (s, 3H, C_q_C*H*_3_), 1.60–1.15 (m, 10H), 0.84 (t, *J* = 6.6 Hz, 3H, CH_3_-C_5_H_11_-CH_2_CO). ^19^F NMR (376 MHz, CDCl_3_): δ −123.29 (dd, *J* = 35.9, 20.9 Hz). HRMS-ESI (*m/z*): [M + Na]^+^ calcd. for C_26_H_35_FN_4_O_5_S_3_Na: 621.1646, found: 621.1650.

### 3.2. Biology

#### 3.2.1. Recombinant Human HDAC1, HDAC2, HDAC3, HDAC6 and HDAC8 Enzymatic Assays

The assays were carried out by Shanghai ChemPartner Co., Ltd (Shanghai, China), according to our previous method [23,24]. Briefly, different concentrations of compounds were incubated with recombinant human HDAC1, HDAC2, HDAC3, HDAC6 and HDAC8 (BPS Biosciences, San Diego, CA, USA) at room temperature for 15 min, which was followed by adding Ac-peptide-AMC substrates to initiate the reaction in Tris-based assay buffer. Reaction mixtures were incubated at room temperature for 60 min in HDAC1, HDAC2, HDAC3 and HDAC6 assays, and were incubated for 240 min in HDAC8 assay. Then, the stop solution containing trypsin was added. The coupled reaction was incubated for another 90 min at 37 °C. Fluorescent AMC released from substrate was measured in SynergyMx (BioTek, Winooski, VT, US) using filter sets as excitation = 355 nm and emission = 460 nm. IC_50_ values were calculated by GraphPad Prism software (7.0 version., GraphPad Software, San Diego, CA, USA).

#### 3.2.2. Cellular Assay

Eca-109, Bel 7402 and U937 cell lines were purchased from ATCC (Manassas, VA, USA), and the other tested cell lines were obtained from the Shanghai Institute for Biological Sciences, Chinese Academy of Sciences (Shanghai, China). MDA-MB-231, Hela, Eca-109 and Bel 7402 cells were cultured in DMEM medium supplemented with 10% heat-inactivated fetal bovine serum, 100 U/mL penicillin and 100 μg/mL streptomycin (Beyotime Biotech, Shanghai, China). A549 cells were cultured in F-12K medium supplemented with 10% heat-inactivated fetal bovine serum, 100 U/mL penicillin and 100 μg/mL streptomycin. HCT116 and SK-OV-3 cells were cultured in McCoy’s 5A medium supplemented with 10% heat-inactivated fetal bovine serum, 100 U/mL penicillin and 100 μg/mL streptomycin. U937 cells were cultured in RPMI-1640 medium supplemented with 10% heat inactivated fetal bovine serum, 100 U/mL penicillin and 100 μg/mL streptomycin. All cells were cultured at 37 °C in humidified air containing 5% CO_2_. According to our previous method [23,24], A549, HCT116, MDA-MB-231, SK-OV-3, Hela, Eca-109, Bel 7403 and U937 (600,000 cells/well) were seeded in 96-well plates and incubated for 24 h before being treated with various concentration of compounds or solvent control. Cells were further incubated for 72 h and then treated with MTT and incubated for another 3 h. The media was then removed and 100 μL DMSO was added to each well. The absorbance at 550 nm was measured by a SpectraMAX340 microplate reader (Molecular Devices, San Jose, CA, USA) with a reference wavelength at 690 nm. Adriamycin was used as a positive control in the assay.

### 3.3. Molecular Modeling Study 

The known crystal complexes of HDAC1, HDAC6, HDAC8 and their ligand (PDB code: 5ICN, 5EDU, 4RN0) were obtained from PDB (http://www.rcsb.org). Molecular docking simulations in the HDACs were run using the MOE 2019 (Molecular Operating Environment, Chemical Computing Group, Montreal, Quebec, Canada) due to its universality and very fast speed. Ligands were prepared with the ChemBio3D Ultra 14.0 (PerkinElmer, Waltham, MA, USA), followed by MM2 energy minimization. Protein structures were also prepared with the MOE, which could automatically add hydrogen atoms to proteins by explicitly considering the protonation state of histidine and optimize the force field. All crystal water, small ligands and cofactors except HEM were removed. After this step, the binding sites were deduced from the known crystal complexes and the ligands were docked to the prepared proteins through flexible docking mode. Top scoring function poses were selected as representative of the simulations and were displayed with Open-Source PyMOLTM 1.8X software (Schrödinger, Ltd, New York, NY, USA).

## 4. Conclusions

Given our previous finding that fluorination at the C18 position of largazole showed good tolerance towards the inhibitory activity and selectivity of HDACs, the current study investigated further modifications on the valine residue in its macrocyclic moiety with S-Me Cysteine or Glycine residue. While the Glycine-modified fluoro analog showed poor activity, the S-Me l-Cysteine-modified analog emerged to be a very potent HDAC inhibitor. Unlike all previously reported C2-modified compounds in the largazole family (including our recent fluoro-largazole analogs) where replacement of the Val residue has failed to provide any potency improvement, the S-Me l-Cysteine-modified analog displayed significantly enhanced (five–nine-fold) inhibition of all the tested HDACs while maintaining the selectivity of HDAC1 over HDAC6, as compared to largazole thiol. Molecular modeling study provided rational explanation and structural evidence for the enhanced activity. This new finding will aid the design of novel potent HDAC inhibitors. An expanded research program is currently under way to investigate largazole analogs bearing similar structural characteristics.

## Figures and Tables

**Table 1:** The preliminary in vitro growth inhibition of several cells (IC_50_, µM) with Largazole and its analog **16c**–**d**.

	IC_50_^a^ (µM)			
	A549	HCT116	MDA-MB-231	SK-OV-3
largazole	0.52 ± 0.17	0.11 ± 0.01	4.75 ± 1.95	0.25 ± 0.17
**16c**	2.92 ± 0.53	1.41 ± 0.54	>10	3.13 ± 0.50.
**16d**	>10	6.62 ± 1.86	>10	>10

^a^ Compounds were tested in at least seven-dose IC_50_ mode in duplicate with three-fold serial dilution.

**Table 2:** The further in vitro growth inhibition of other cells (IC_50_, µM) with Largazole and **16c–d**.

	IC_50_^a^ (µM)			
	Hela	Eca-109	Bel 7402	U937
largazole	0.17 ± 0.01	0.10 ± 0.00	0.17 ± 0.02	0.02 ± 0.00
**16c**	0.10 ± 0.01	0.09 ± 0.02	0.25 ± 0.07	0.08 ± 0.02

^a^ Compounds were tested in at least seven-dose IC_50_ mode in duplicate with three-fold serial dilution.

**Table 3:**
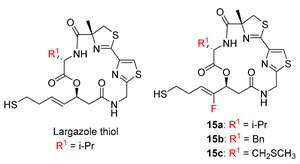
In vitro inhibition of human histone deacetylases (HDACs) with the free thiols of largazole and its two analogs.

			IC_50_ (nM) ^a^			Selectivity (HDAC1/6)
	HDAC1	HDAC2	HDAC3	HDAC8	HDAC6	
**15a** ^b^	4.39 ± 0.17	21.0 ± 0.3	39.2 ± 2.3	8.38 ± 2.46	300 ± 5	68
**15b** ^b^	4.24 ± 0.38	16.3 ± 0.4	37.1 ± 0.8	17.5 ± 4.2	338 ± 17	80
**15c**	0.27 ± 0.02	1.33 ± 0.02	2.33 ± 0.15	0.44 ± 0.16	23.9 ± 0.2	89
largazole thiol	2.01 ± 0.01	9.49 ± 0.19	14.4 ± 0.2	3.75 ± 0.20	121 ± 5	60

^a^ Compounds were tested in 10-dose IC_50_ mode in duplicate with three-fold serial dilution. ^b^ The data were previously reported by us in [23].

## Supplementary Materials

The supporting information is available online at https://www.mdpi.com/1660-3397/18/7/344/s1. Figures S1–S3: ^1^H, ^13^C and ^19^F NMR spectra of compound 8, Figure S4: ^1^H NMR spectra of compound 11, Figures S5–S7: ^1^H, ^13^C and ^19^F NMR spectra of compound 9, Figures S8 and S9: ^1^H and ^13^C NMR spectra of compound 10c, Figures S10–S12: ^1^H, ^13^C and ^19^F NMR spectra of compound 12c; Figures S13–S15: ^1^H, ^13^C and ^19^F NMR spectra of compound 12d, Figures S16–S18: ^1^H, ^13^C and ^19^F NMR spectra of compound 14c, Figures S19–S21: ^1^H, ^13^C and ^19^F NMR spectra of compound 14d, Figures S22–S25: ^1^H, COSY, ^13^C and ^19^F NMR spectra of compound 15c, Figures S26–S28: ^1^H, ^13^C and ^19^F NMR spectra of compound 16c, Figures S29–S31: ^1^H, ^13^C and ^19^F NMR spectra of compound 16d, Figures S32–S39: The concentration-response curves of Largazole and 16c in the cellular assays, Figures S40–S42: The concentration-response curves of Largazole thiol in HDACs assays, Figures S43–S45: The concentration-response curves of 15c in HDACs assays.
