# Supplementary material for: Unexpected Enhancement of HDACs Inhibition by MeS Substitution at C-2 Position of Fluoro Largazole
Source: Mar Drugs. 2020 Jun 30;18(7):344. doi: 10.3390/md18070344 (PMC7401273; doi:10.3390/md18070344)
Supplement: Supplementary file 1 [file marinedrugs-18-00344-s001.pdf]

Supporting Information

Title: Unexpected enhancement of HDACs inhibition by MeS substitution at C-2 position of fluoro largazole

Authors: Bingbing Zhang [1,†], Zhu-Wei Ruan [1,†], Dongdong Luo [2,†], Yueyue Zhu [1], Tingbo Ding [1], Qiang Sui [3] and Xinsheng Lei [1, 4*]

Affiliations:
[1] School of Pharmacy, Fudan University, 826 Zhangheng Road, Pudong Zone, Shanghai 201203, China.
[2] School of Medicine and Pharmacy, Ocean University of China, Qingdao, Shandong Province 26603, China.
[3] China State Institute of Pharmaceutical Industry, No. 285 Gebaini Road, Pudong Zone, Shanghai 201203, China..
[4] Key Laboratory of Synthetic Chemistry of Natural Substances, Shanghai Institute of Organic Chemistry, Chinese Academy of Sciences, Shanghai 200032, China.
***** Correspondence: leixs@fudan.edu.cn; Tel.: +86-021-51980128
[†] These authors contributed equally to the work.

Contact email: leixs@fudan.edu.cn

Table of Contents

1. General experimental P3
2. ^{1}H, ^{13}C and ^{19}F NMR spectra of compound **8**.. P4-5
3. ^{1}H NMR spectra of compound **11**.. P6
4. ^{1}H, ^{13}C and ^{19}F NMR spectra of compound **9** P7-8
5. ^{1}H and ^{13}C NMR spectra of compound **10c**.. P9
6. ^{1}H, ^{13}C and ^{19}F NMR spectra of compound **12c**... P10-11
7. ^{1}H, ^{13}C and ^{19}F NMR spectra of compound **12d**... P12-13
8. ^{1}H, ^{13}C and ^{19}F NMR spectra of compound **14c**... P14-15
9. ^{1}H, ^{13}C and ^{19}F NMR spectra of compound **14d**... P16-17
10. ^{1}H, COSY, ^{13}C and ^{19}F NMR spectra of compound **15c**.. P18-19
11. ^{1}H, ^{13}C and ^{19}F NMR spectra of compound **16c**... P20-21
12. ^{1}H, ^{13}C and ^{19}F NMR spectra of compound **16d**... P22-23
13. The concentration-response curves of Largazole and **16c** in the cellular assays…………P24-26
14. The concentration-response curves of Largazole thiol in HDACs assays………………..P27
15. The concentration-response curves of **15c** in HDACs assays……………………………P28

Experimental Section

General Methods. The chemicals and reagents were purchased from Acros, Alfa Aesar, and National Chemical Reagent Group Co. Ltd., P. R. China, and used without further purification. Anhydrous solvents (THF, MeOH, DMF, CH_2Cl_2, and CH_3CN) used in the reactions were dried and freshly distilled before use. All the reactions were carried out under Ar atmosphere, otherwise stated else. The progress of the reactions was monitored by TLC (silica-coated glass plates) and visualized under UV light, and by using iodine or phosphomolybdic acid. Melting points were measured on a SGW X-4 microscopy melting point apparatus without correction. ^{1}H NMR and ^{13}C NMR spectra were recorded either on a 400 MHz Varian Instrument at 25 °C or 600 MHz Bruke Instrument at 25 °C, using TMS as an internal standard, respectively. Multiplicity is tabulated as s for singlet, d for doublet, dd for doublet of doublet, t for triplet, and m for multiplet. HRMS spectra were recorded on Finnigan-Mat-95 mass spectrometer, equipped with ESI source.

(S,Z)-1-((R)-4-benzyl-2-thioxothiazolidin-3-yl)-4-fluoro-3-hydroxy-7-(tritylthio)hept-4-en-1-one (8).

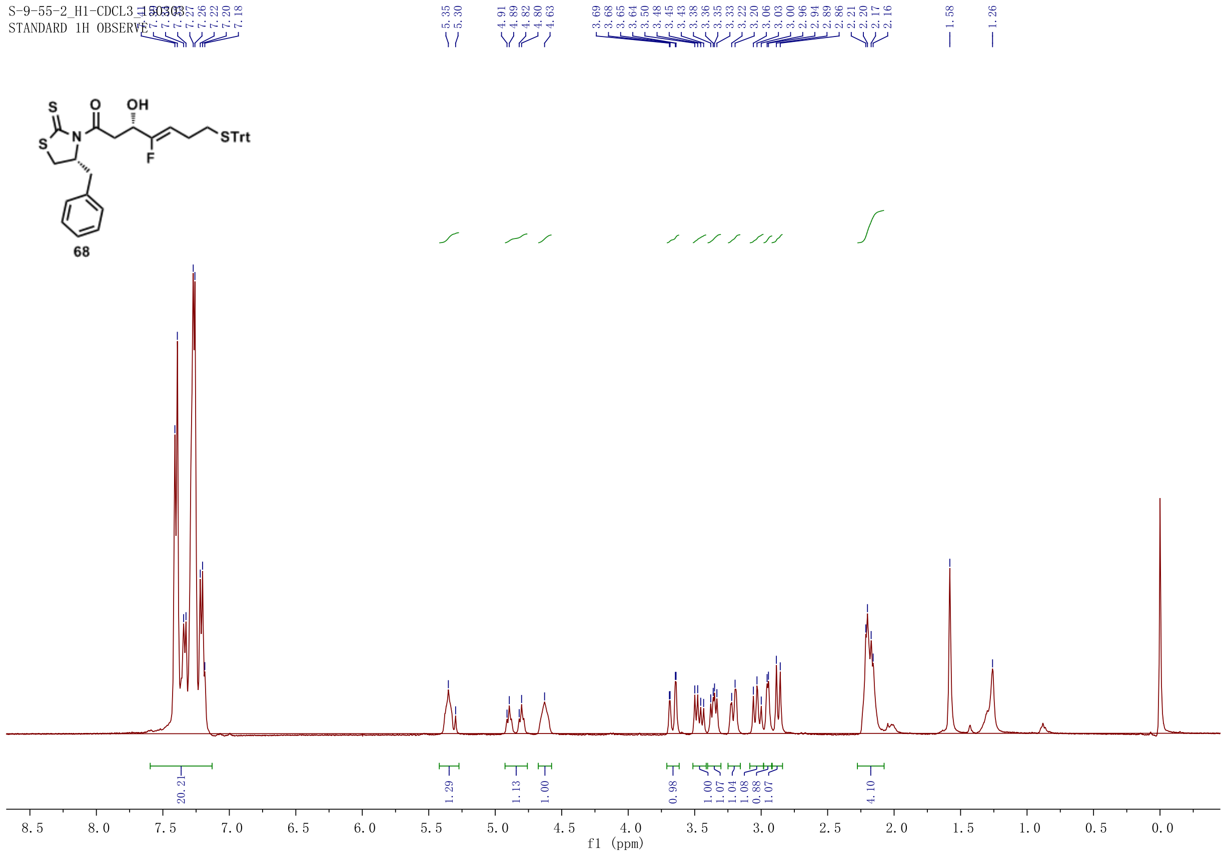

Figure S1. ^{1}H spectra of compound **8**

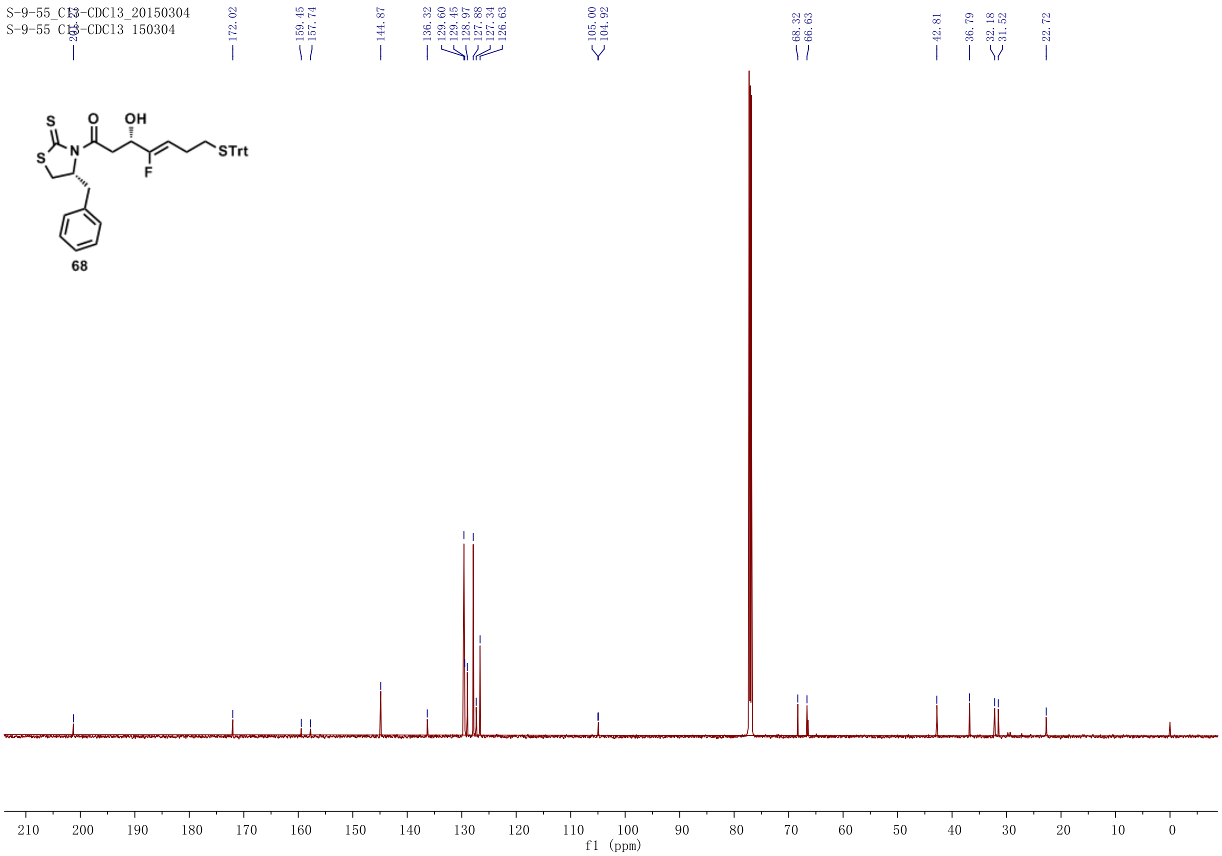

Figure S2. ^{13}C spectra of compound **8**

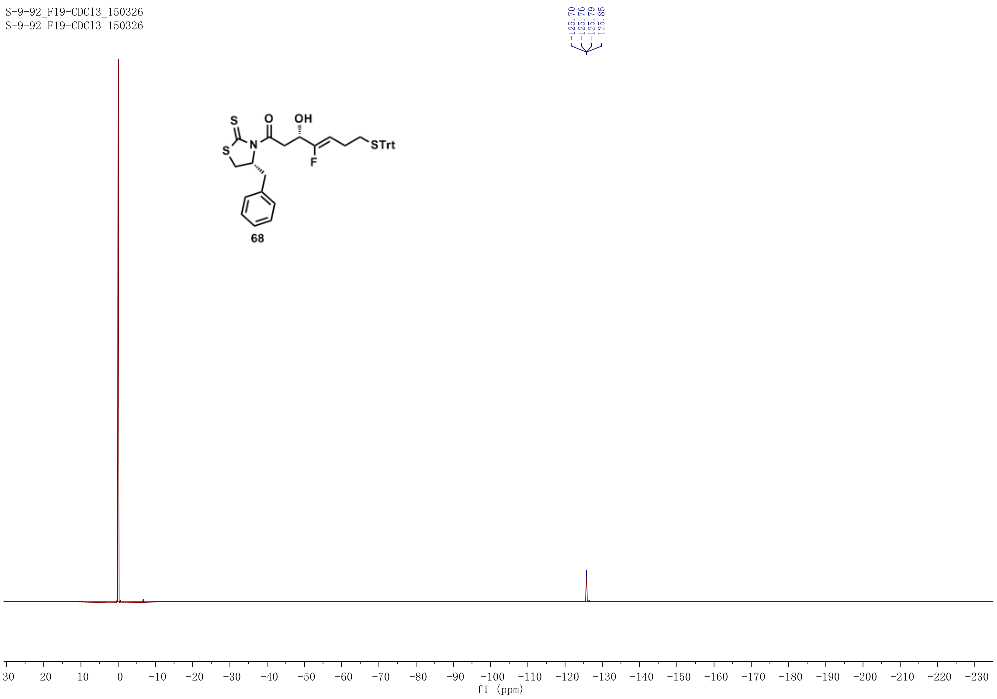

Figure S3. ^{19}F spectra of compound **8**

(R)-2'-(((tert-butoxycarbonyl)amino)methyl)-4-methyl-4,5-dihydro-[2,4'-bithiazole]-4-carboxylic acid (11)

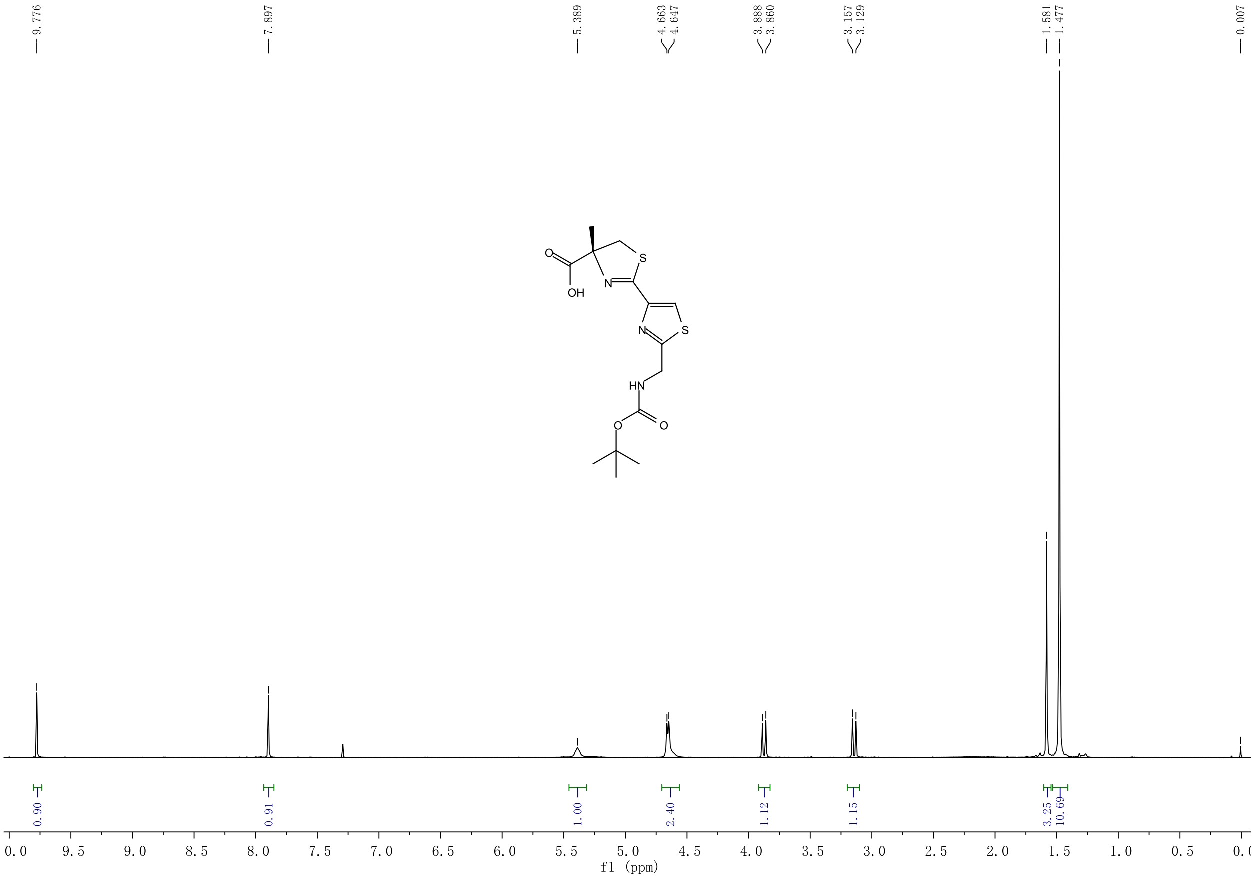

Figure S4. ^{1}H spectra of compound **11**

2-(trimethylsilyl)ethyl (S,Z)-4-fluoro-3-hydroxy-7-(tritylthio)hept-4-enoate (9)

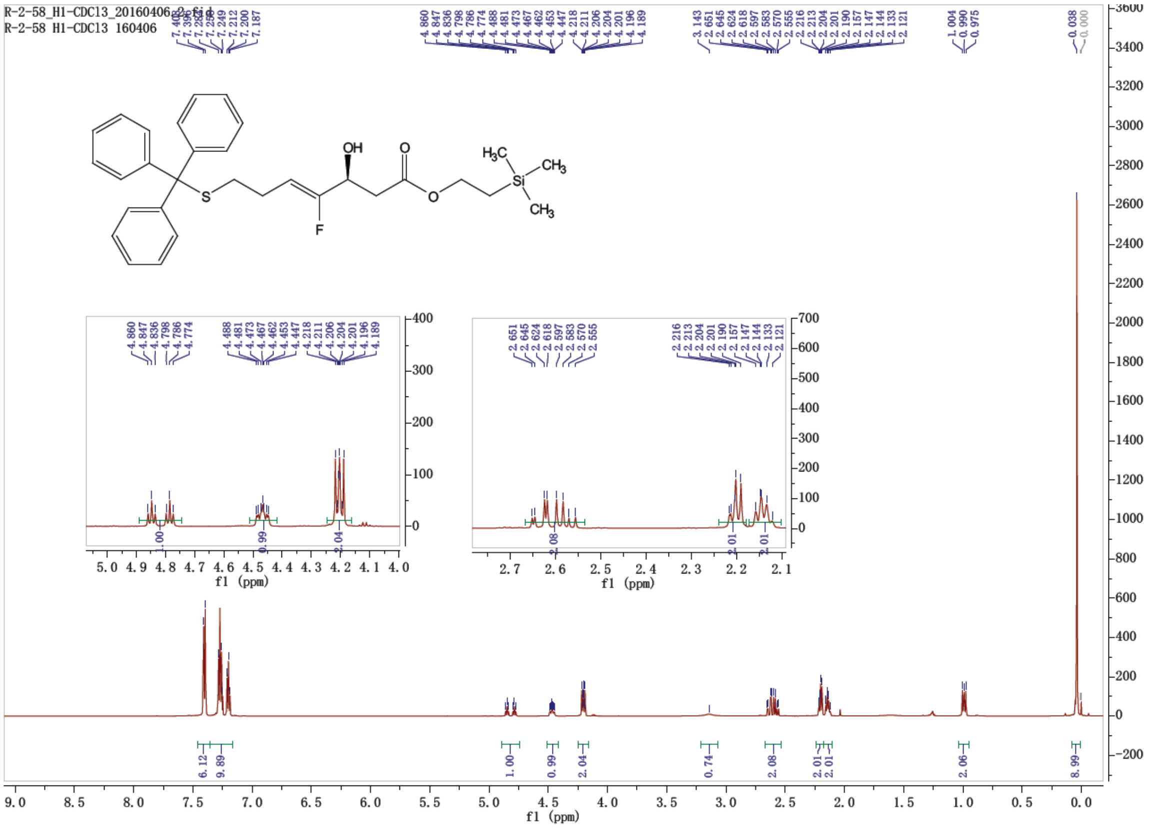

Figure S5. ^{1}H spectra of compound **9**

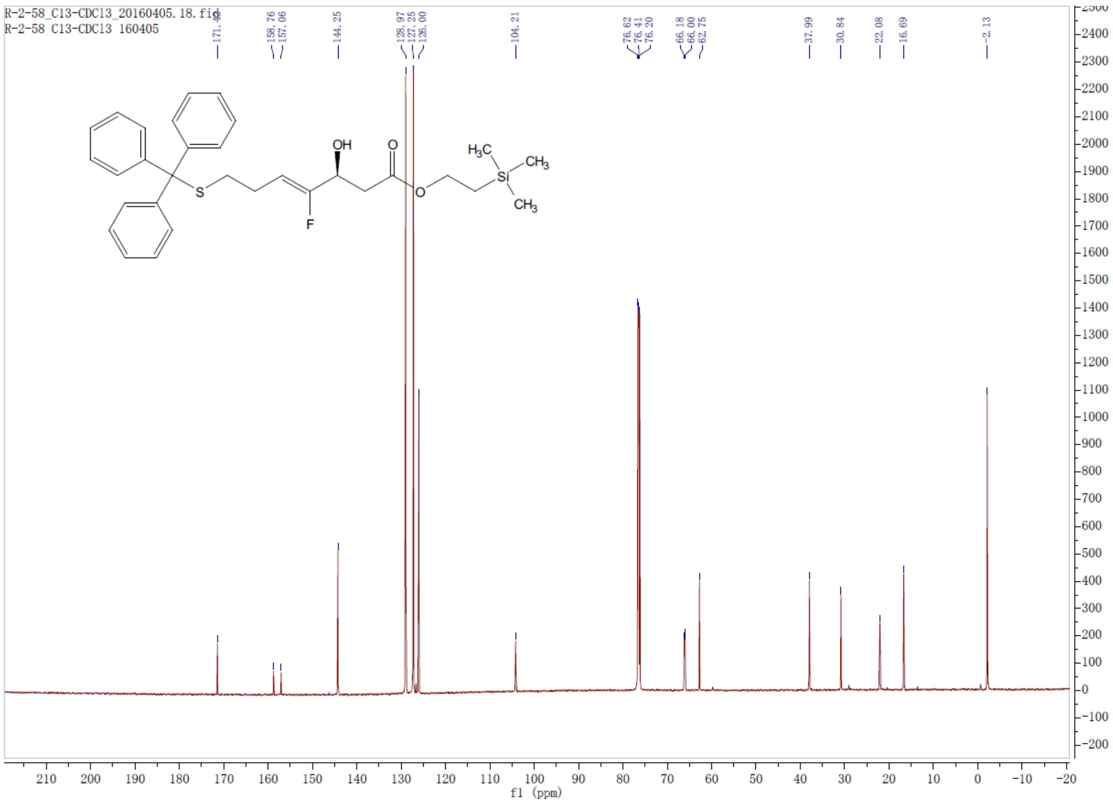

Figure S6. ^{13}C spectra of compound **9**

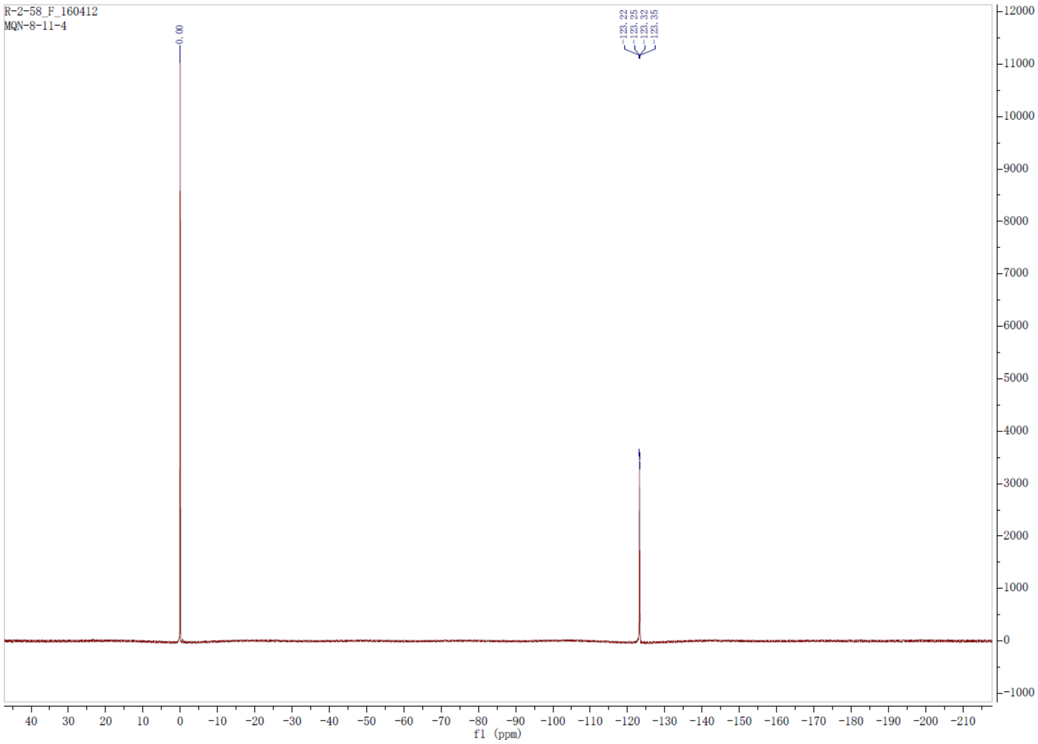

Figure S7. ^{19}F spectra of compound **9**

2-(trimethylsilyl)ethyl (S,Z)-3-((N-(((9H-fluoren-9-yl)methoxy)carbonyl)-S-methyl-L-cysteinyl)oxy)-4-fluoro-7-(tritylthio)hept-4-enoate (10c).

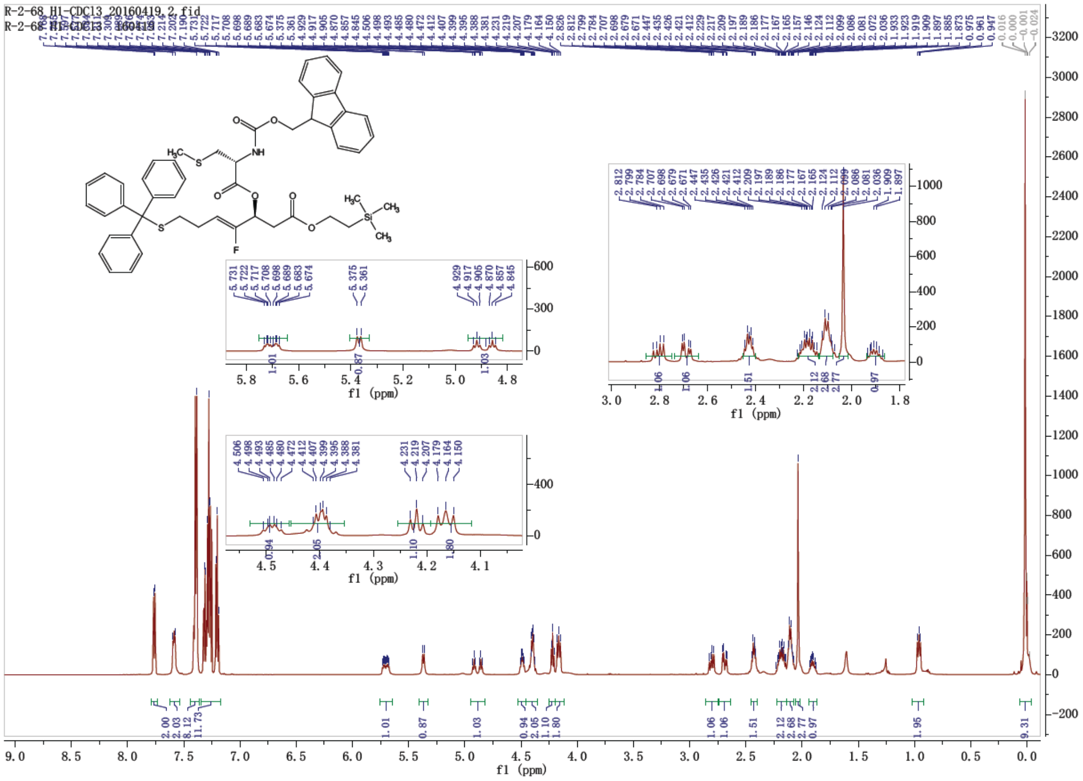

Figure S8. ^{1}H spectra of compound **10c**

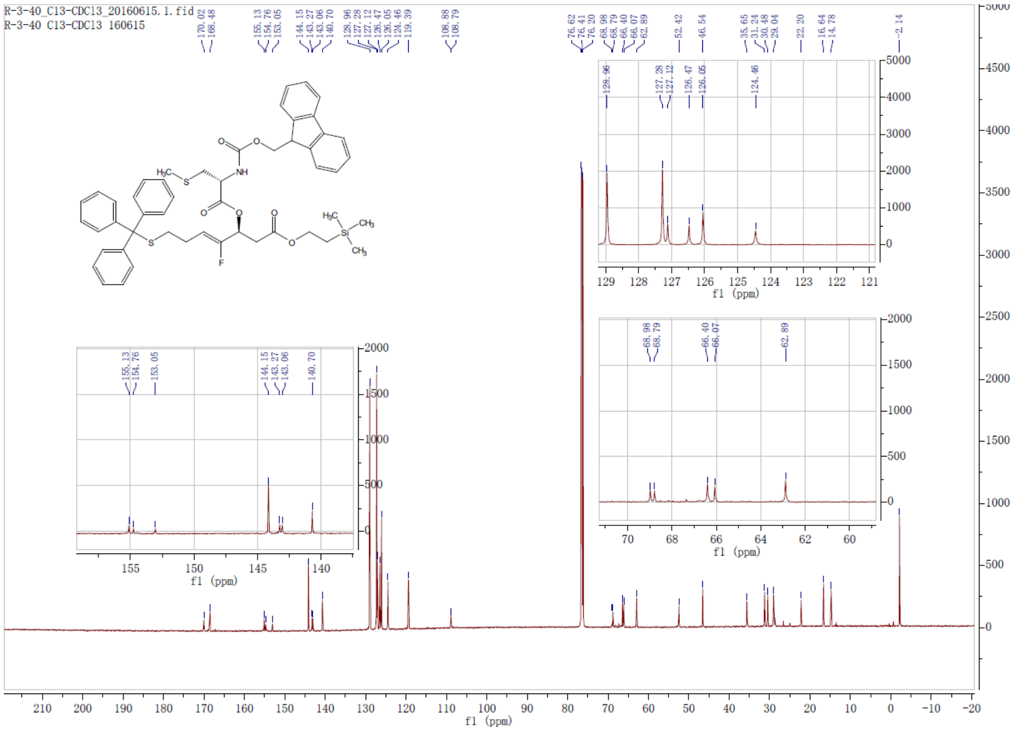

Figure S9. ^{13}C spectra of compound **10c**

2-(trimethylsilyl)ethyl (S,Z)-3-((N-((R)-2′-(((tert-butoxycarbonyl)amino)methyl)-4-methyl-4,5-dihydro-[2,4′-bithiazole]-4-carbonyl)-S-methyl-L-cysteinyl)oxy)-4-fluoro-7-(tritylthio)hept-4-enoate ***(12c)***

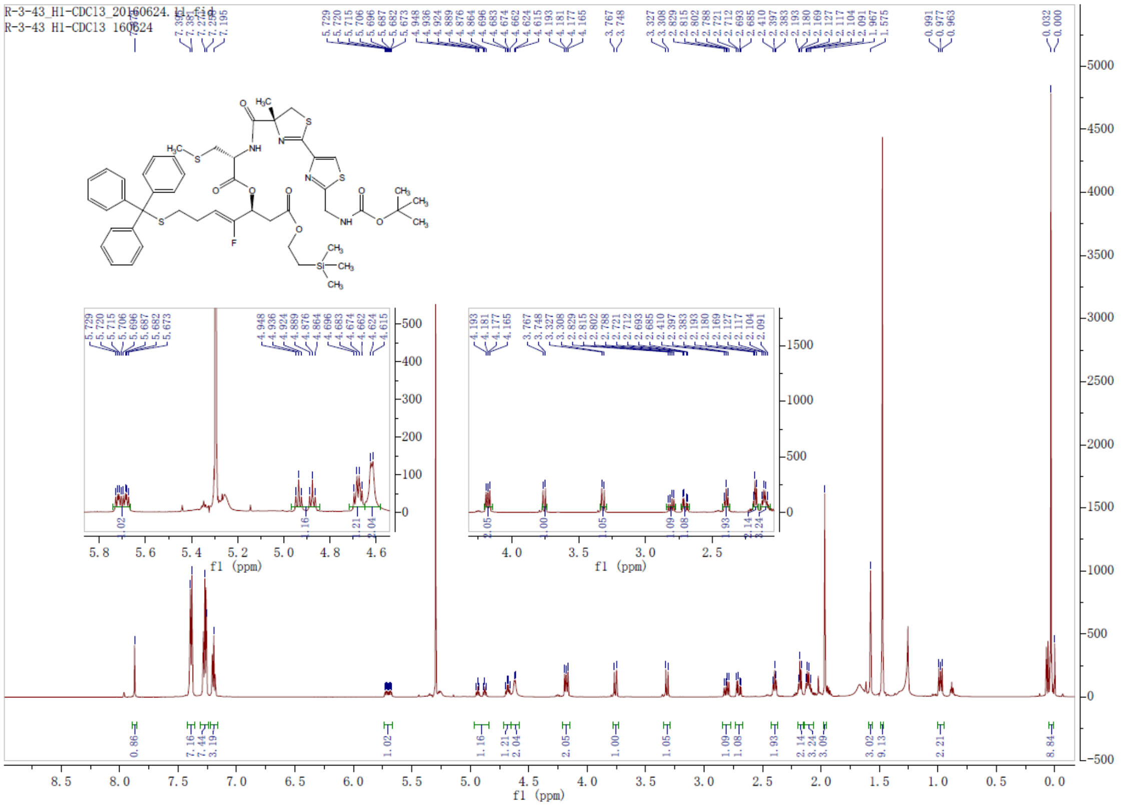

Figure S10. ^{1}H spectra of compound **12c**

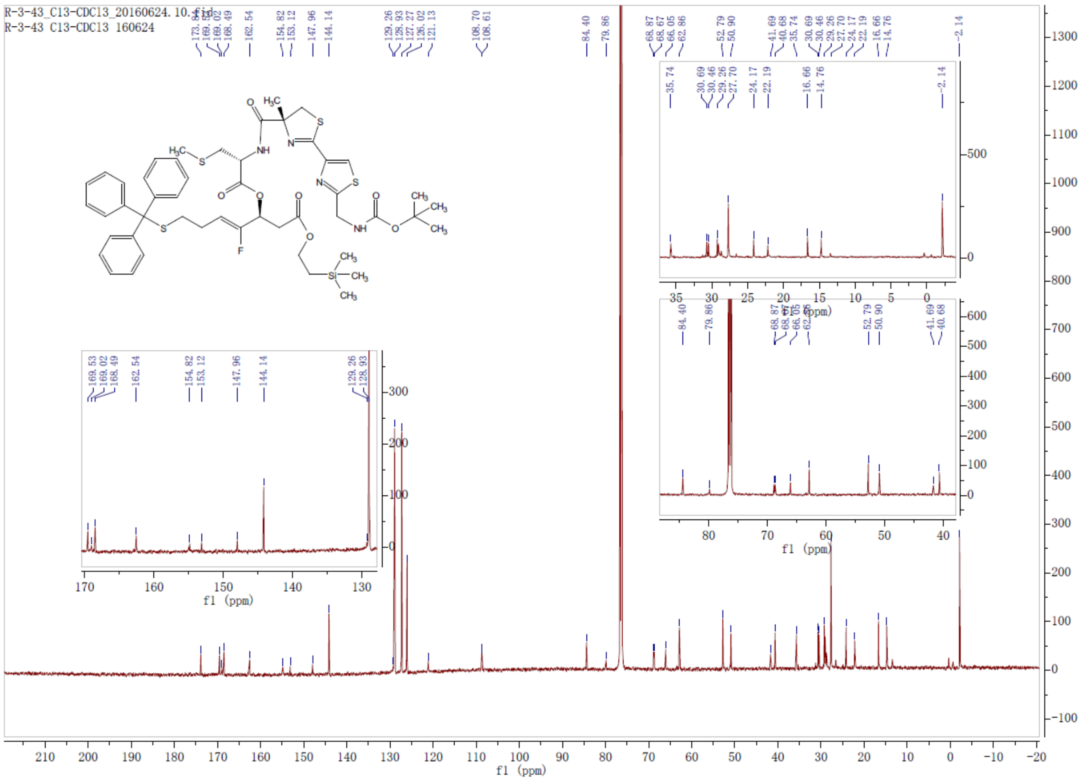

Figure S11. ^{13}C spectra of compound **12c**

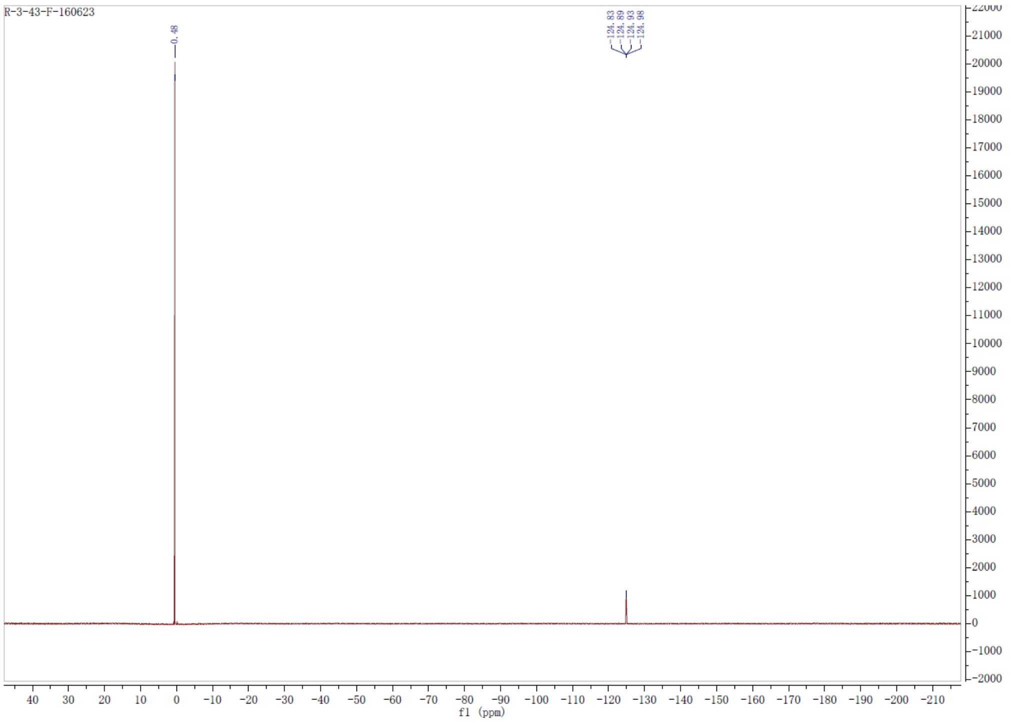

Figure S12. ^{19}F spectra of compound **12c**

*2-(trimethylsilyl)ethyl (S,Z)-3-((((R)-2'-(((tert-butoxycarbonyl)amino)methyl)-4-methyl-4,5-dihydro-[2,4'-bithiazole]-4-carbonyl)glycyl)oxy)-4-fluoro-7-(tritylthio)hept-4-enoate (**12d**)*

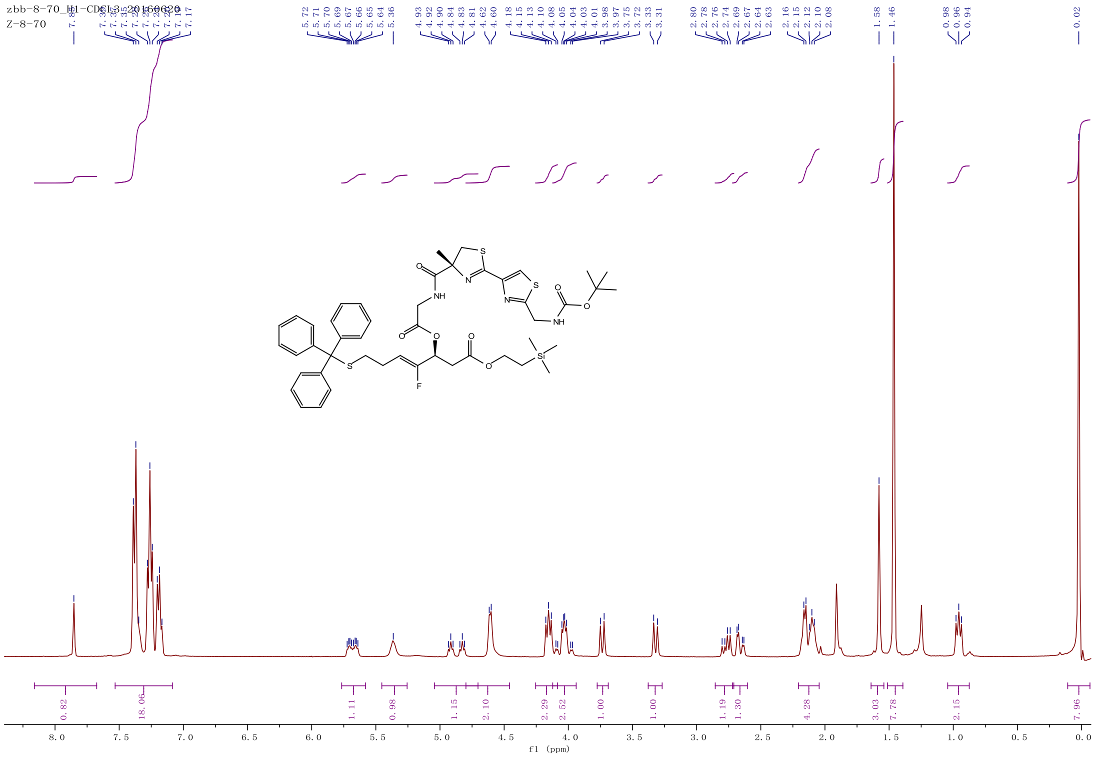

Figure S13. ^{1}H spectra of compound **12d**

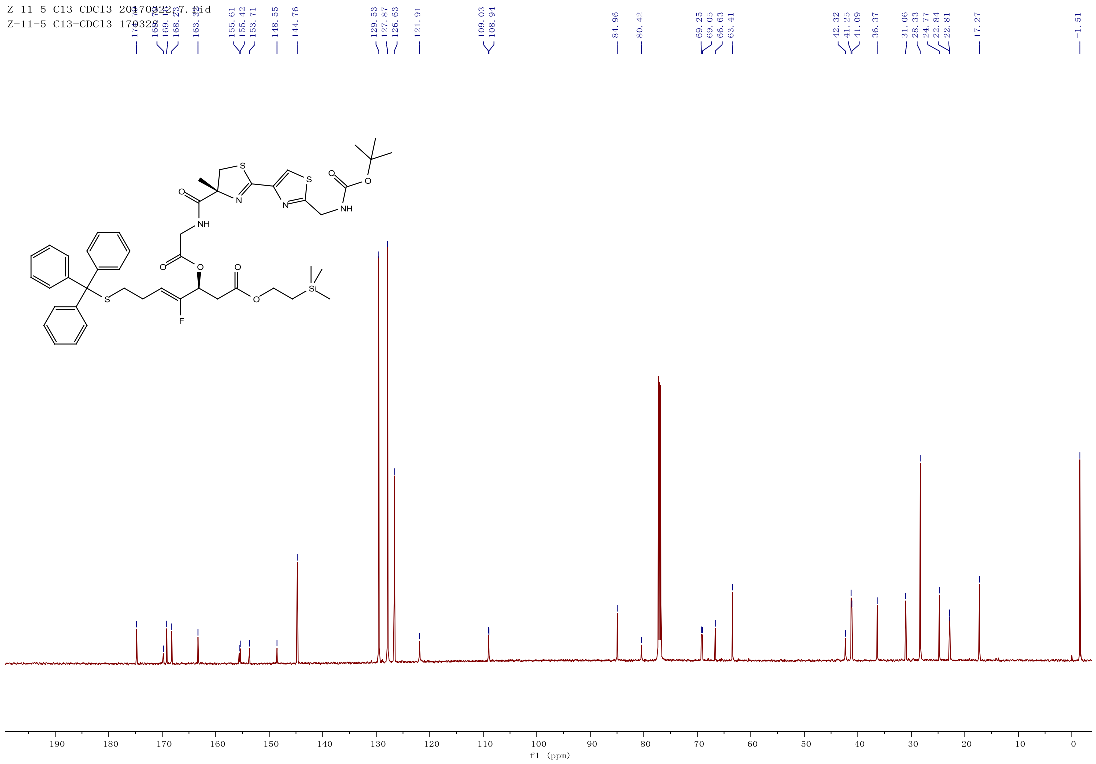

Figure S14.^{13}C spectra of compound **12d**

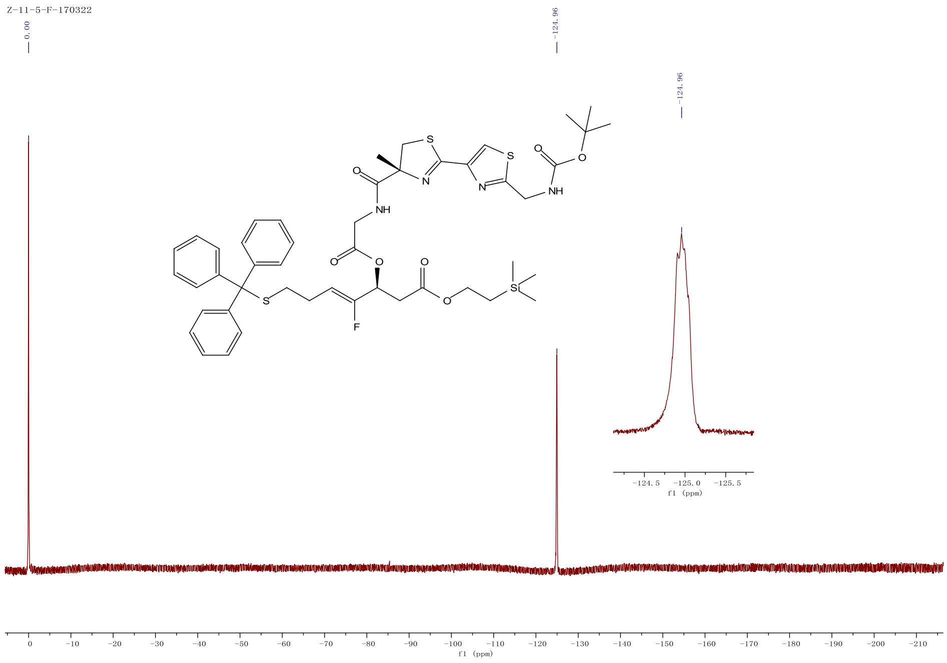

Figure S15. ^{19}F spectra of compound **12d**

*(1^{2}Z,2^{2}Z,2^{4}R,5R,8S)-8-((Z)-1-fluoro-4-(tritylthio)but-1-en-1-yl)-2^4-methyl-5-((methylthio)methyl)-2^4,2^5-dihydro-7-oxa-4,11-diaza-1(4,2),2(2,4)-dithiazolacyclododecaphane-3,6,10-trione (**14c**).*

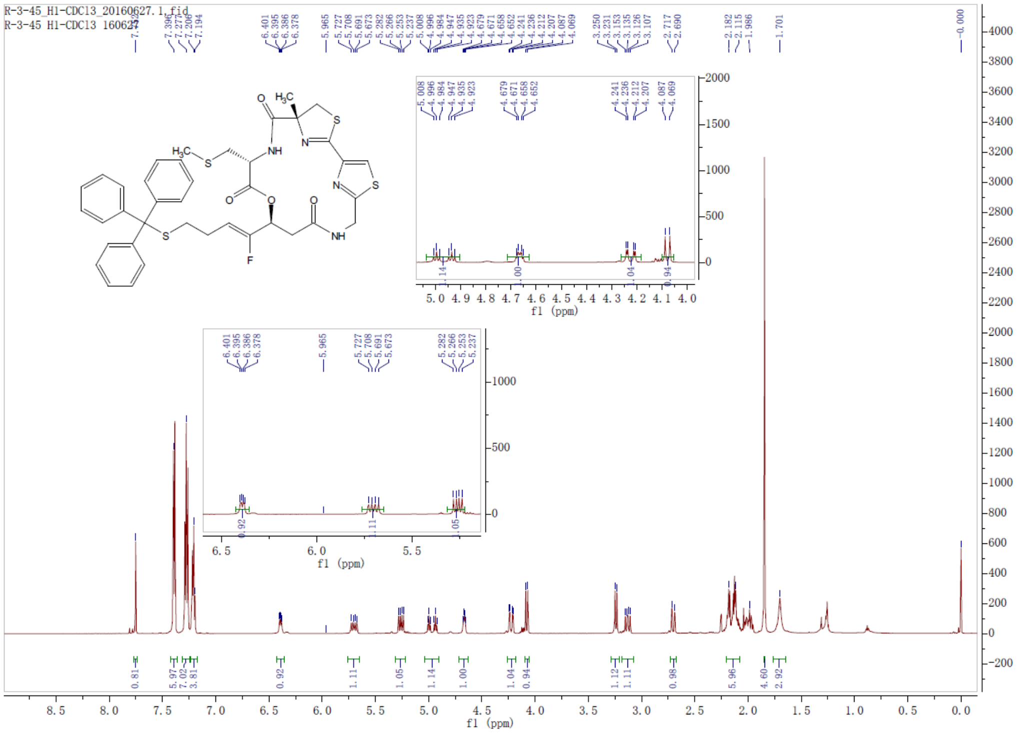

Figure S16. ^{1}H spectra of compound **14c**

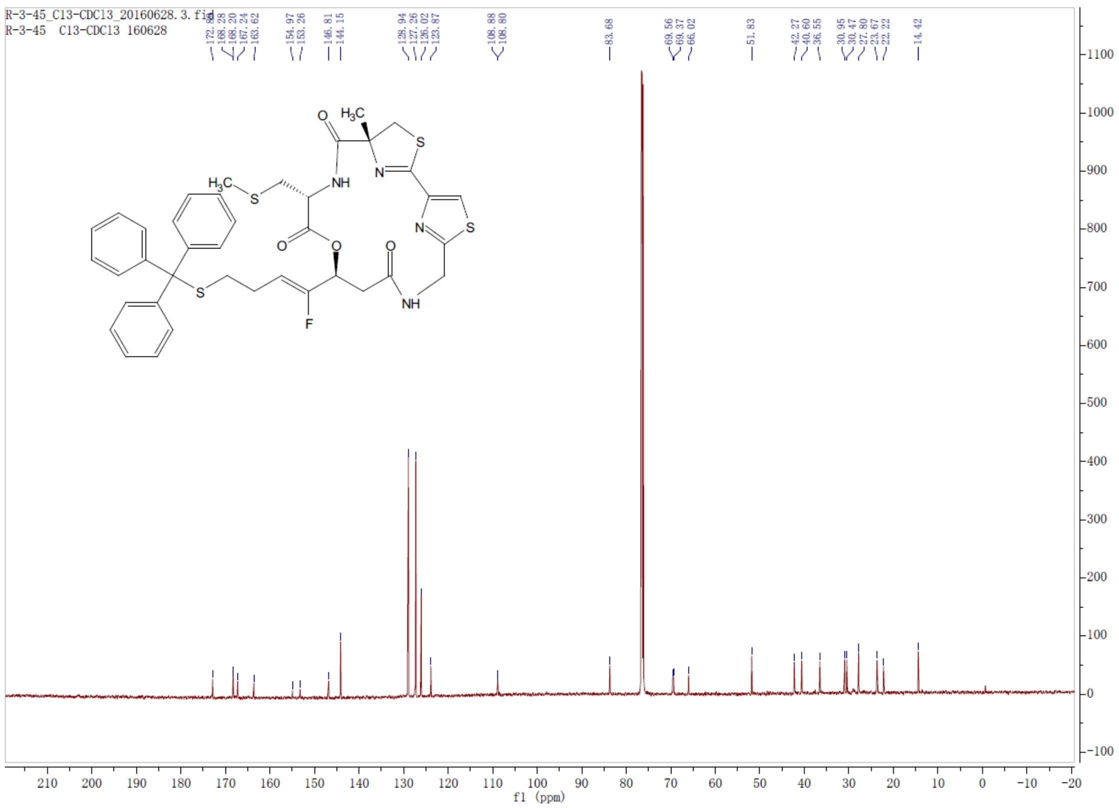

Figure S17. ^{13}C spectra of compound **14c**

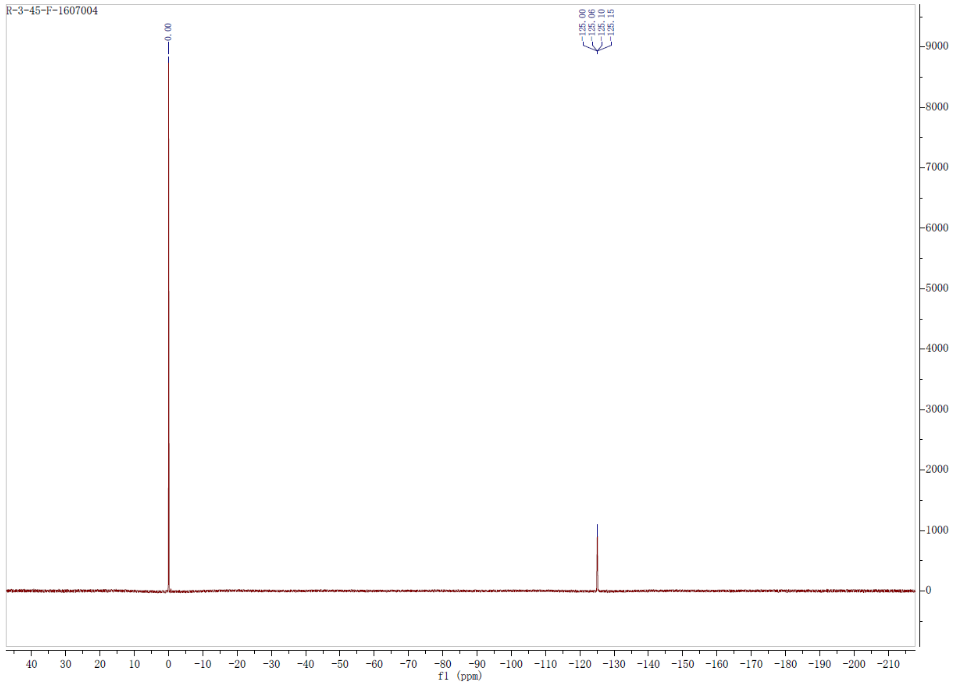

Figure S18. ^{19}F spectra of compound **14c**

*(1^2Z,2^2Z,2^4R,8S)-8-((Z)-1-fluoro-4-(tritylthio)but-1-en-1-yl)-2^4-methyl-2^4,2^5-dihydro-7-oxa-4,11-diaza-1(4,2),2(2,4)-dithiazolacyclododecaphane-3,6,10-trione (**14d**).*

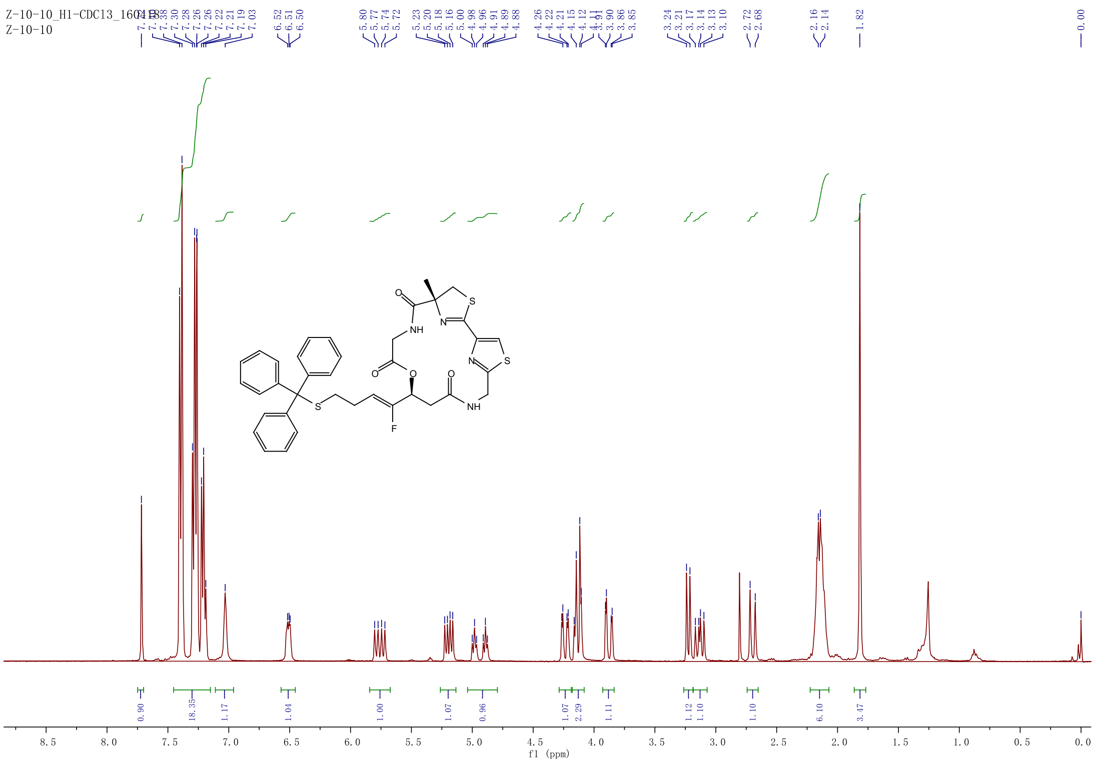

Figure S19. ^{1}H spectra of compound **14d**

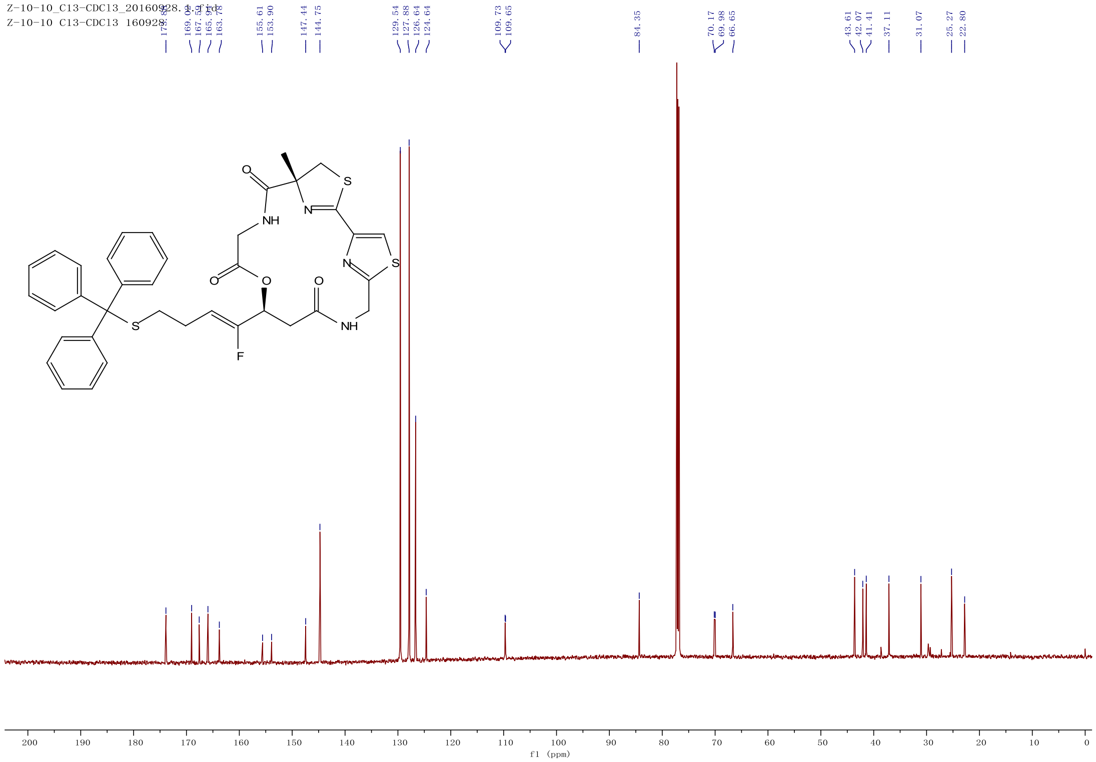

Figure S20.^{13}C spectra of compound **14d**

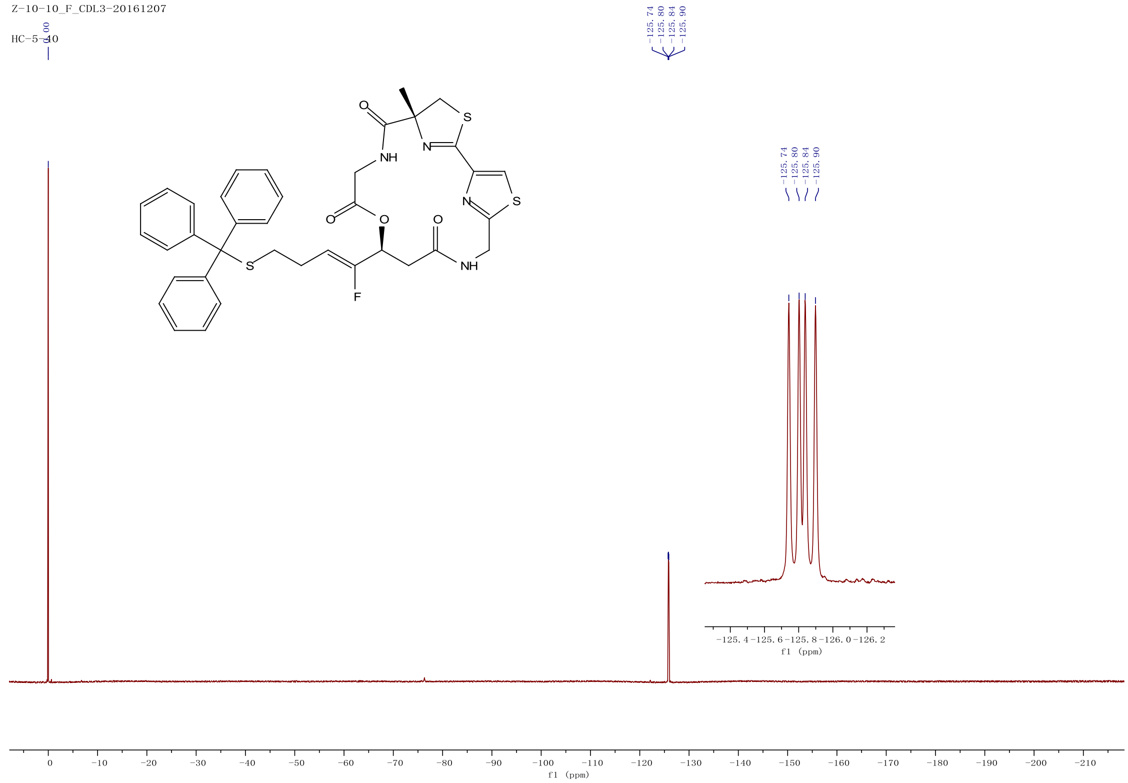

Figure S21. ^{19}F spectra of compound **14d**

*($1^{2}Z$,$2^{2}Z$,$2^{4}R$,8S)-8-((Z)-1-fluoro-4-mercaptobut-1-en-1-yl)-2^{4}-methyl-5-((methylthio)methyl)-2^{4},2^{5}-dihydro-7-oxa-4,11-diaza-1(4,2),2(2,4)-dithiazolacyclododecaphane-3,6,10-trione (**15c**).*

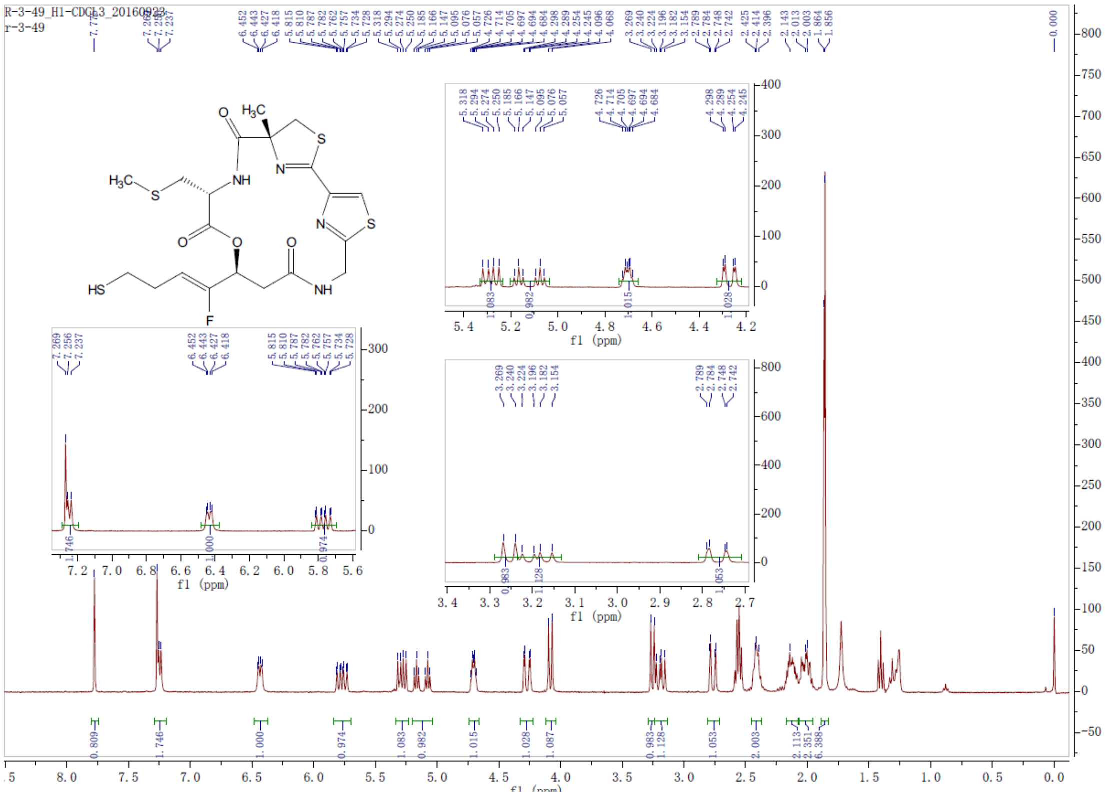

Figure S22. ^{1}H spectra of compound **15c**

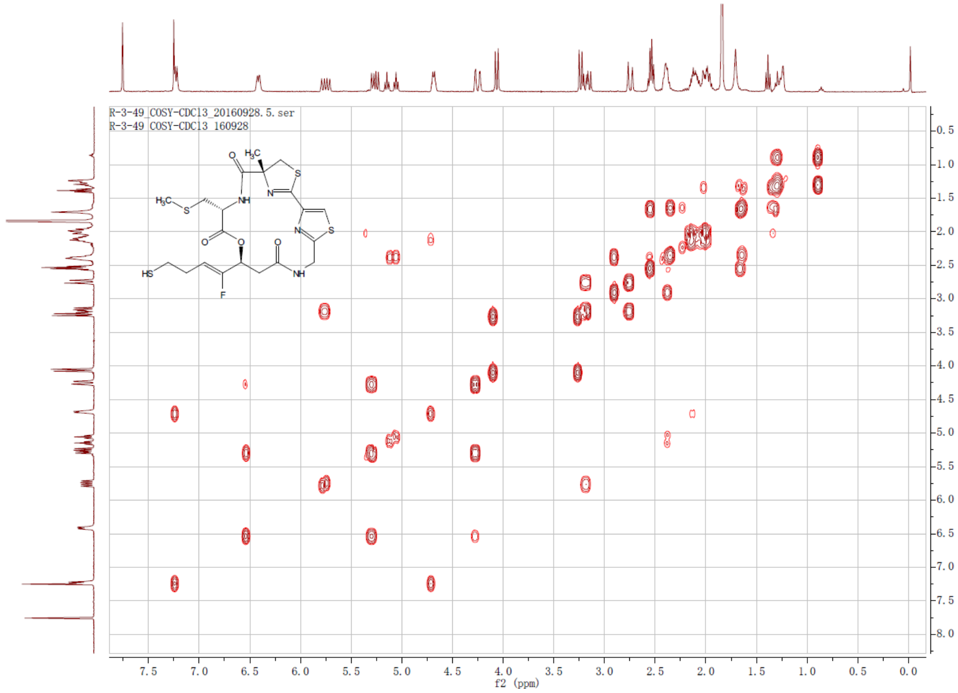

Figure S23. COSY spectra of compound **15c**

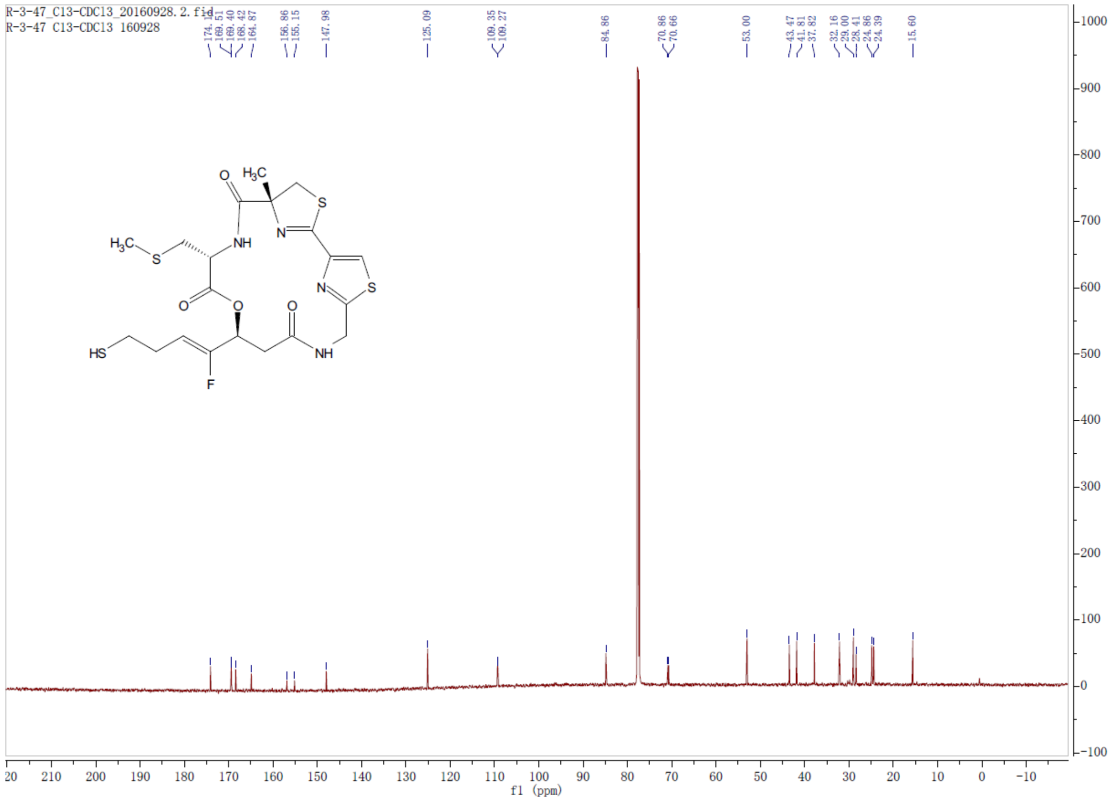

Figure S24. ^{13}C spectra of compound **15c**

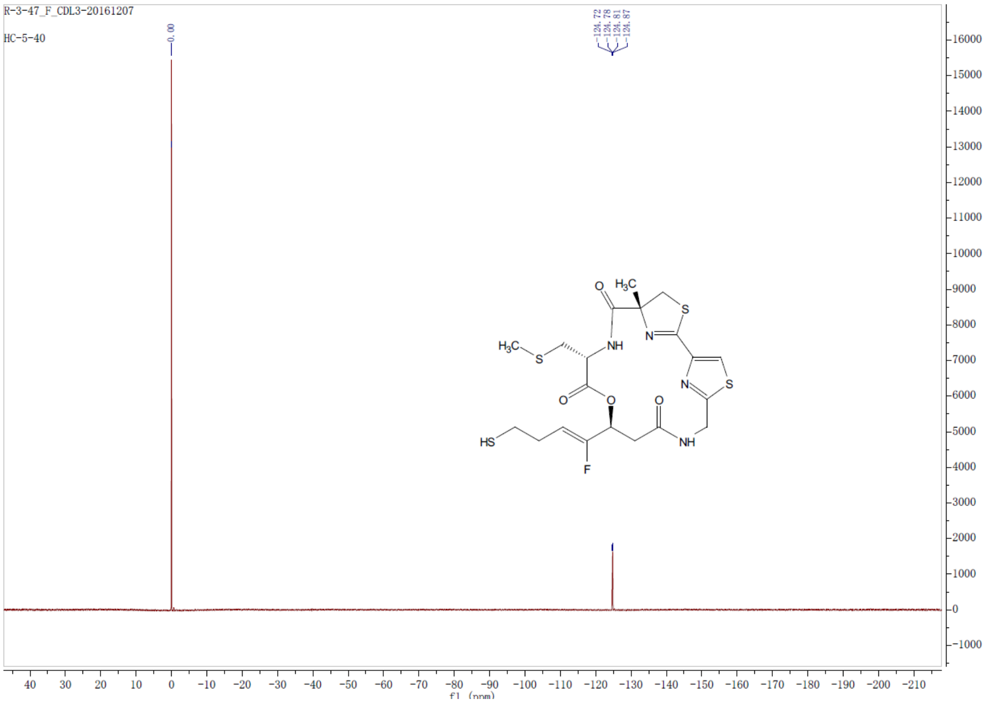

Figure S25. ^{19}F spectra of compound **15c**

*S-((Z)-4-fluoro-4-(($1^{2}Z,2^{2}Z,2^{4}R$,5R,8S)-2^{4}-methyl-5-((methylthio)methyl)-3,6,10-trioxo-2^{4},2^{5}-dihydro-7-oxa-4,11-diaza-1(4,2),2(2,4)-dithiazolacyclododecaphane-8-yl)but-3-en-1-yl) octanethioate (**16c**).*

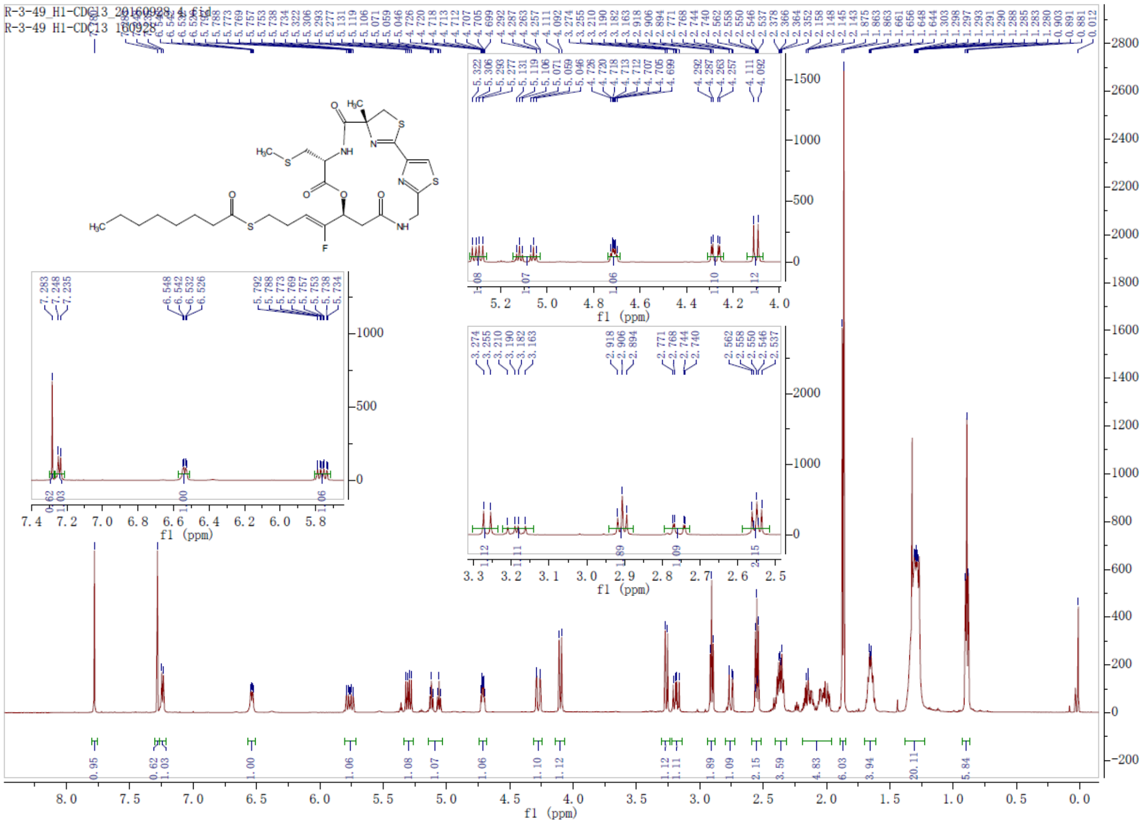

Figure S26. ^{1}H spectra of compound **16c**

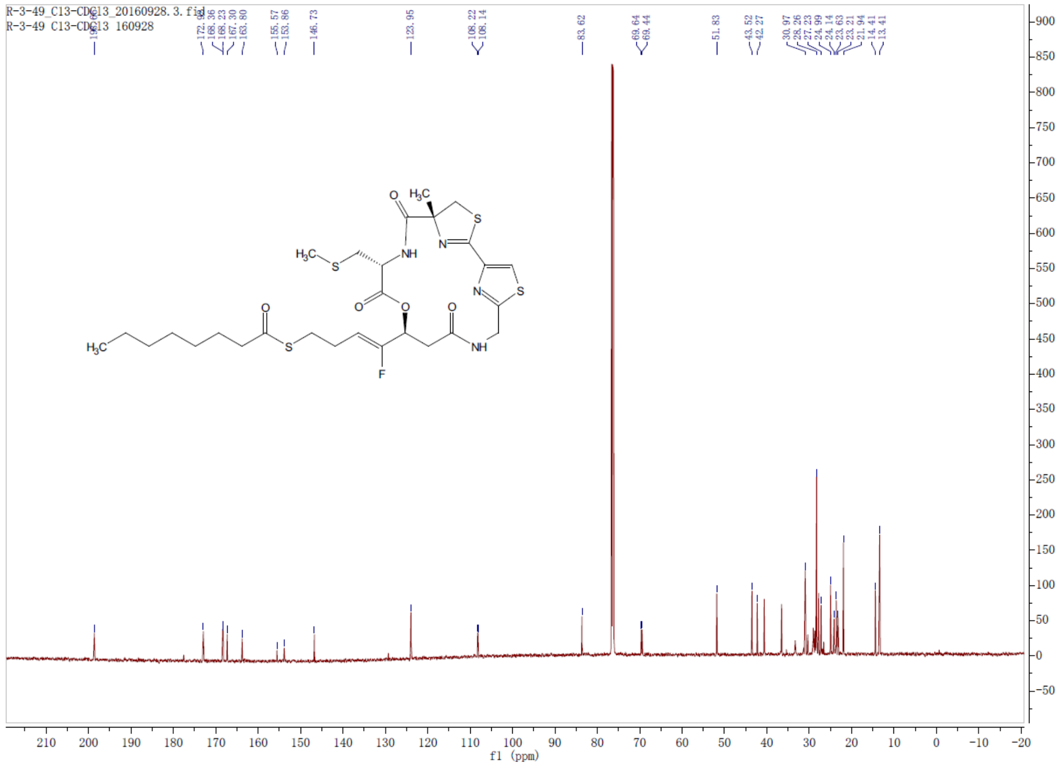

Figure S27. ^{13}C spectra of compound **16c**

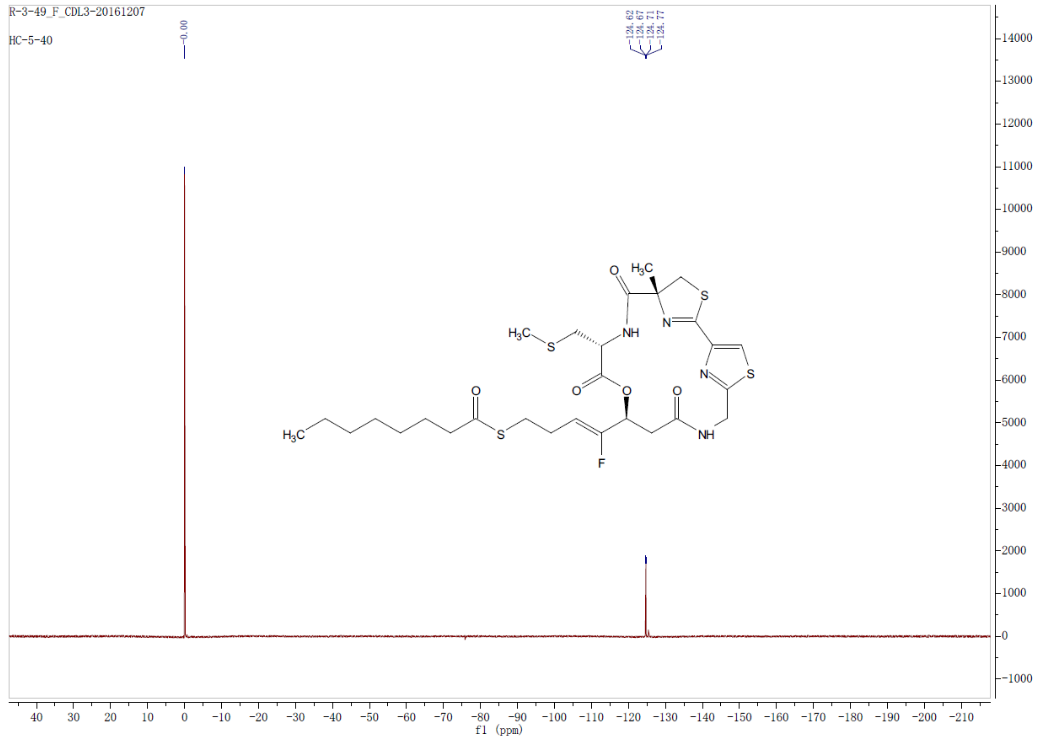

Figure S28. ^{19}F spectra of compound **16c**

*S-((Z)-4-fluoro-4-((1²Z,2²Z,2⁴R,8S)-2⁴-methyl-3,6,10-trioxo-2⁴,2⁵-dihydro-7-oxa-4,11-diaza-1(4,2),2(2,4)-dithiazolacyclododecaphane-8-yl)but-3-en-1-yl) octanethioate (**16d**).*

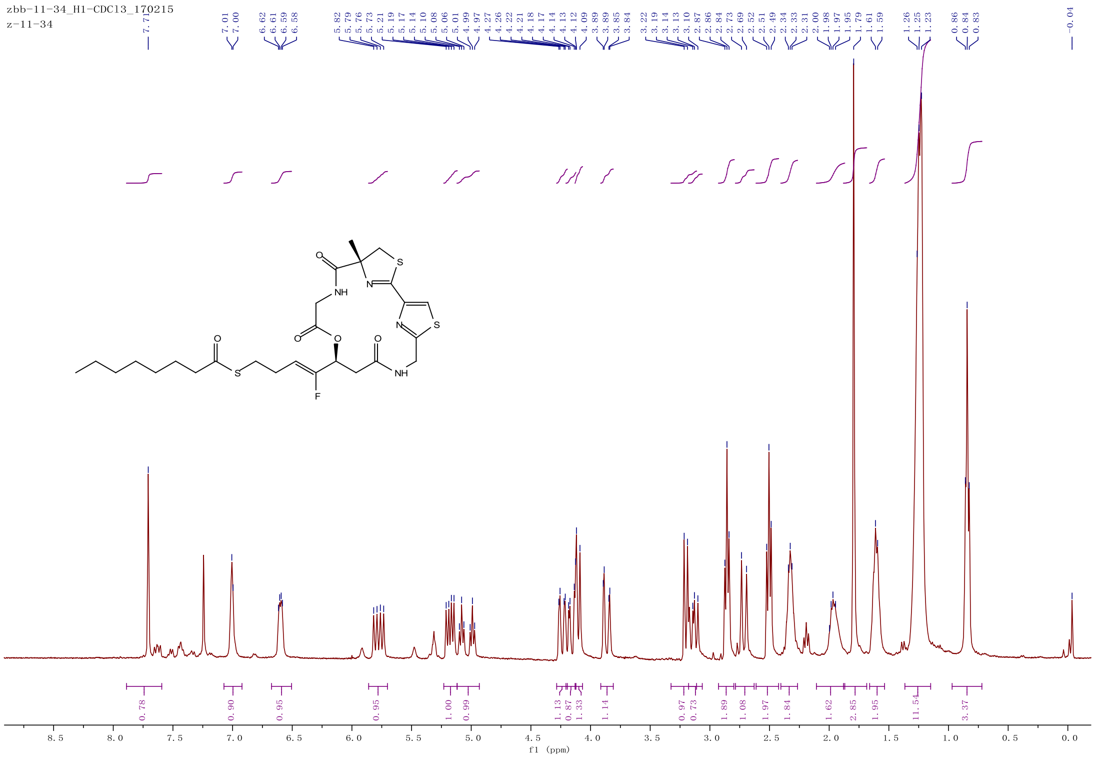

Figure S29.^{1}H spectra of compound **16d**

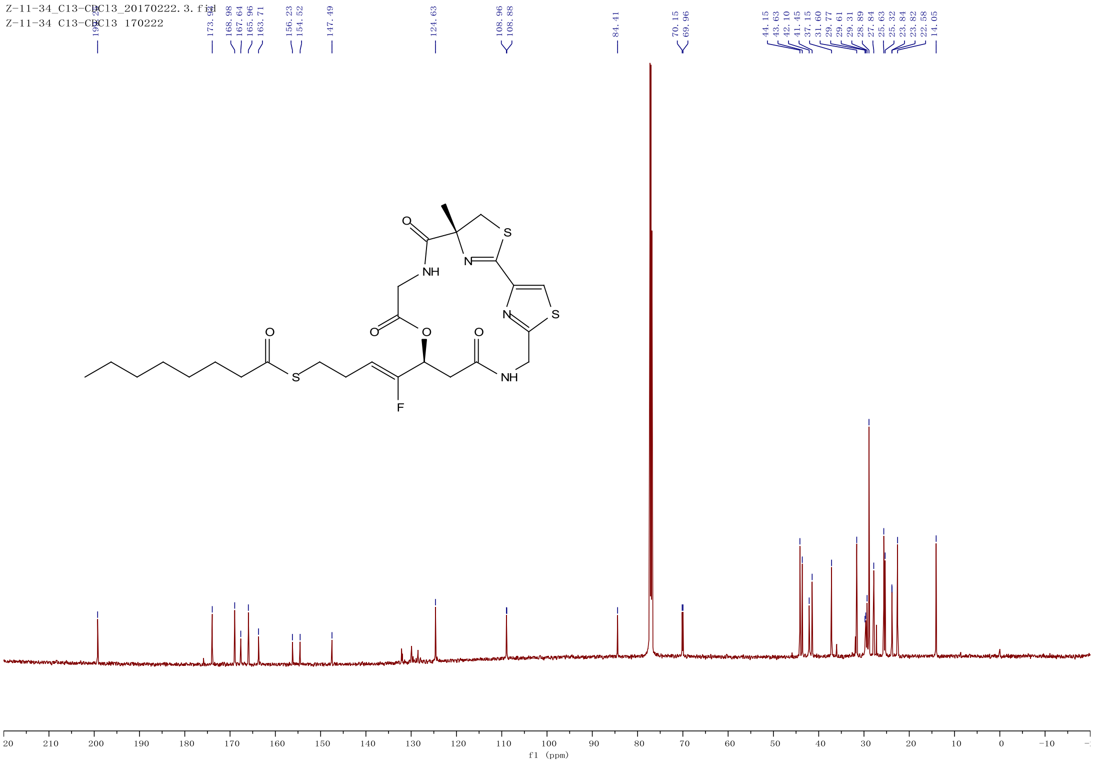

Figure S30. ^{13}C spectra of compound **16d**

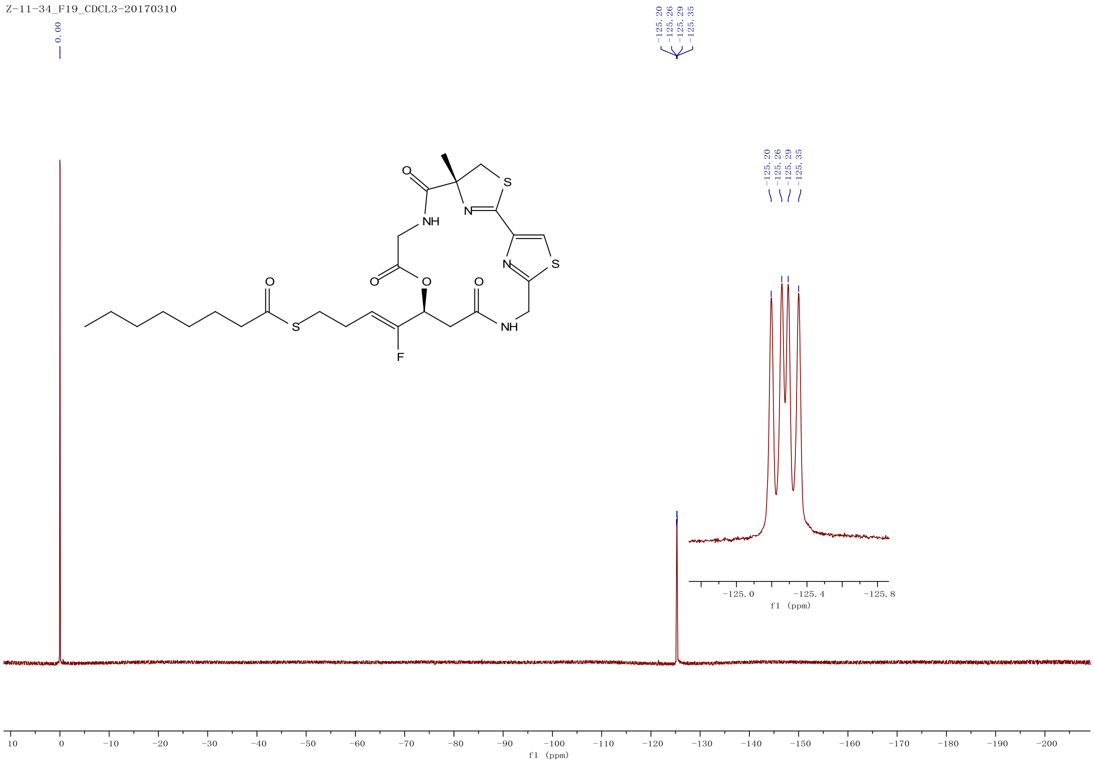

Figure S31. ^{19}F spectra of compound **16d**

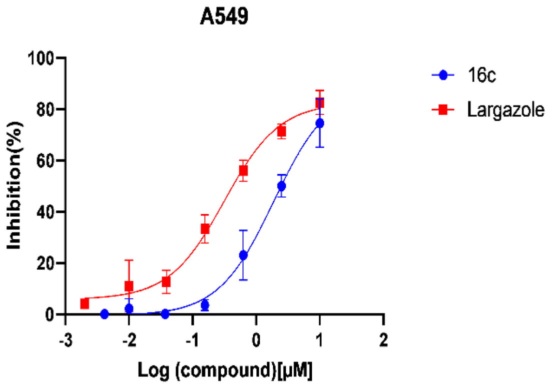

Figure S32. **The concentration-response curves of Largazole and 16c in the growth inhibition of A549 cells.**

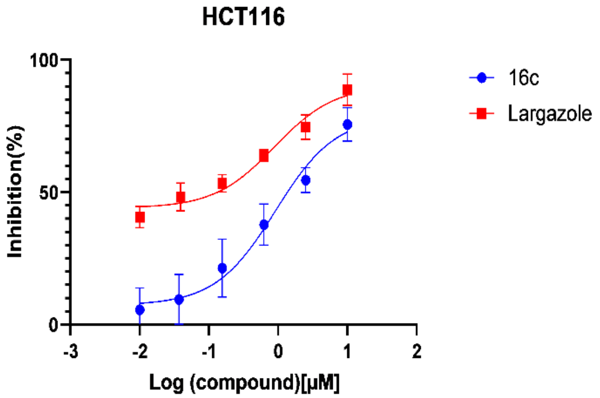

Figure S33. **The concentration-response curves of Largazole and 16c in the growth inhibition of HCT116 cells.**

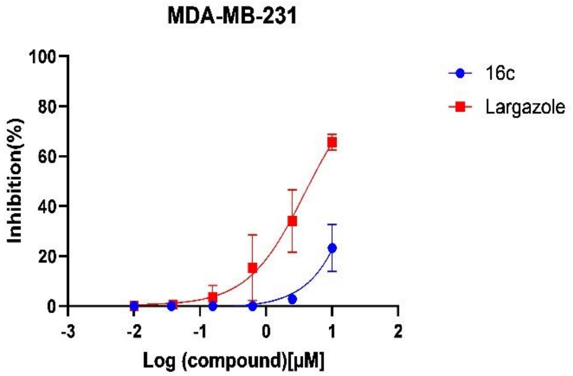

Figure S34. **The concentration-response curves of Largazole and 16c in the growth inhibition of MDA-MB-231 cells**

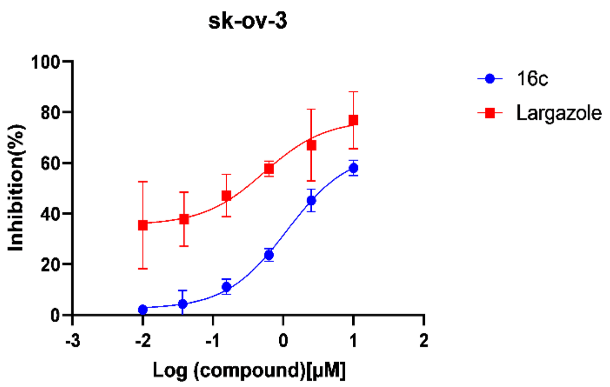

Figure S35. **The concentration-response curves of Largazole and 16c in the growth inhibition of SK-OV-3 cells**

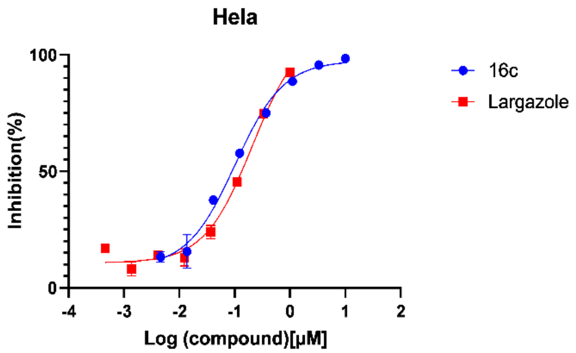

Figure S36. **The concentration-response curves of Largazole and 16c in the growth inhibition of Hala cells**

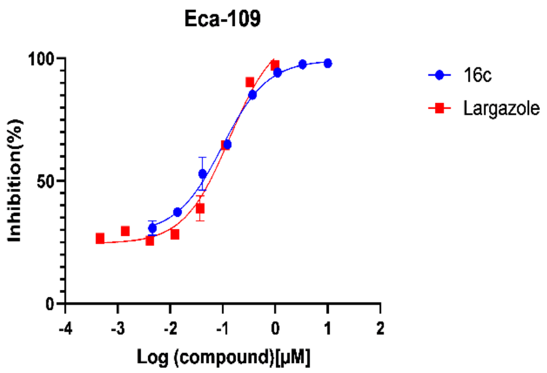

Figure S37. **The concentration-response curves of Largazole and 16c in the growth inhibition of Eca-109 cells**

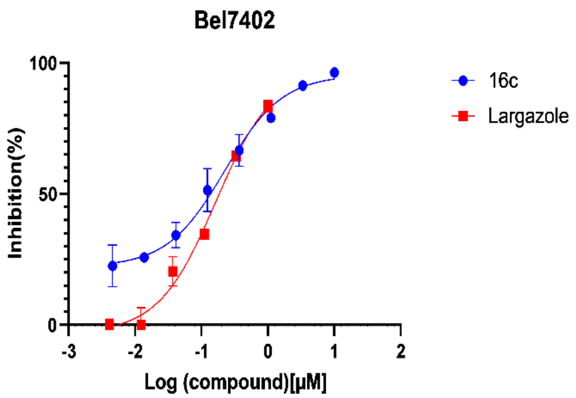

Figure S38. **The concentration-response curves of Largazole and 16c in the growth inhibition of Bel7402 cells**

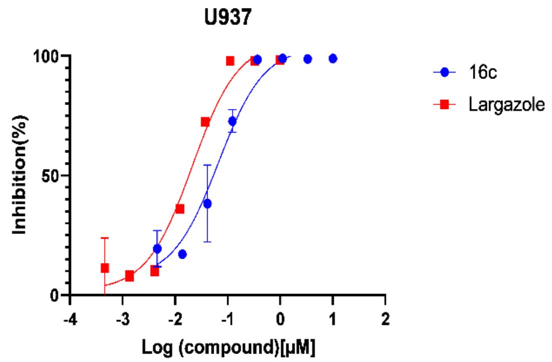

Figure S39. **The concentration-response curves of Largazole and 16c in the growth inhibition of U973 cells**

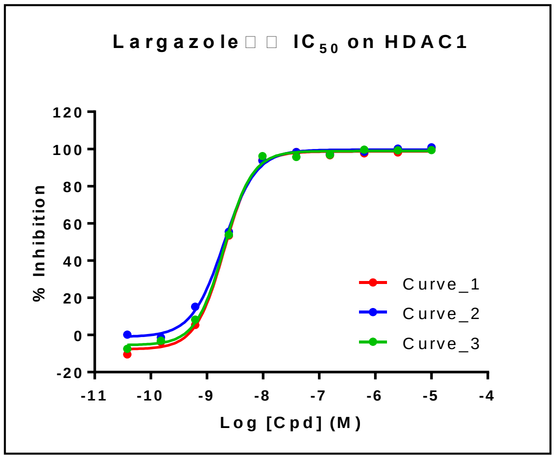

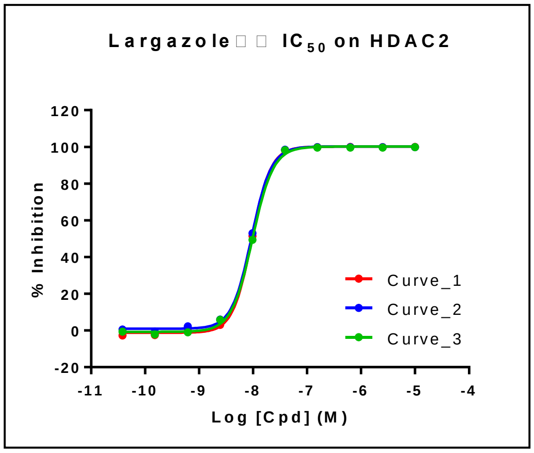

Figure S40. **The concentration-response curves of largazole thiol in HDAC1-2 assays (**Note: Largazole thiol has been named as Largazole □□ in the figures.**)**

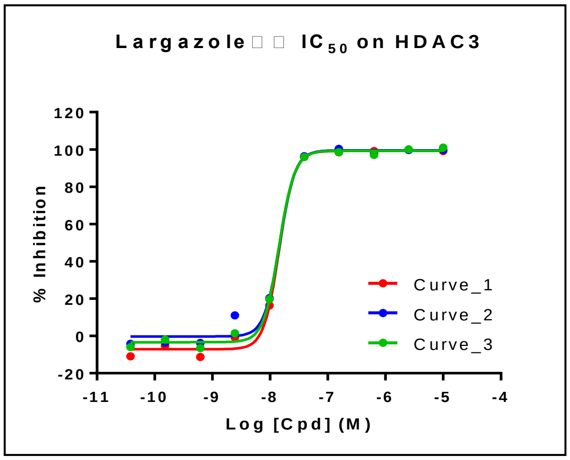

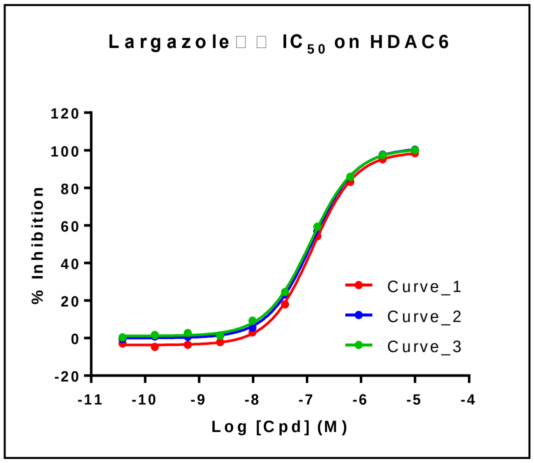

Figure S41. **The concentration-response curves of largazole thiol in HDAC3 and HDAC6 assays (**Note: Largazole thiol has been named as Largazole □□ in the figures.**)**

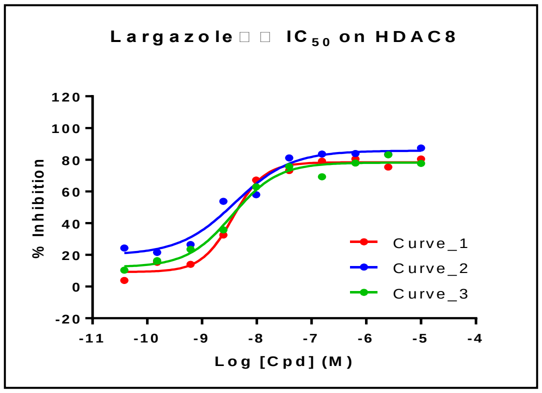

Figure S42. **The concentration-response curves of largazole thiol in HDAC8 assays (**Note: Largazole thiol has been named as Largazole □□ in the figure.**)**

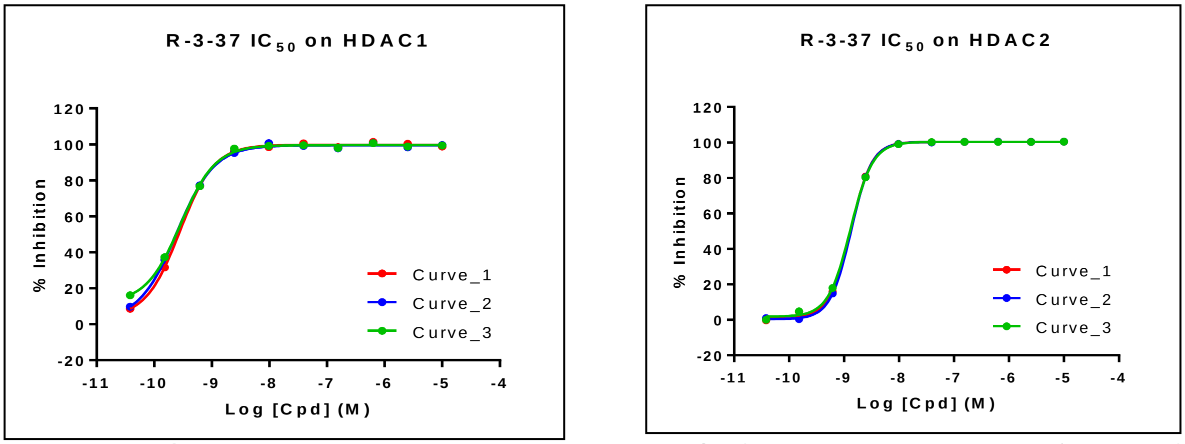

Figure S43. **The concentration-response curves of 15c in HDAC1-2 assays (**Note: **15c** has been named as **R-3-37** in the figures.**)**

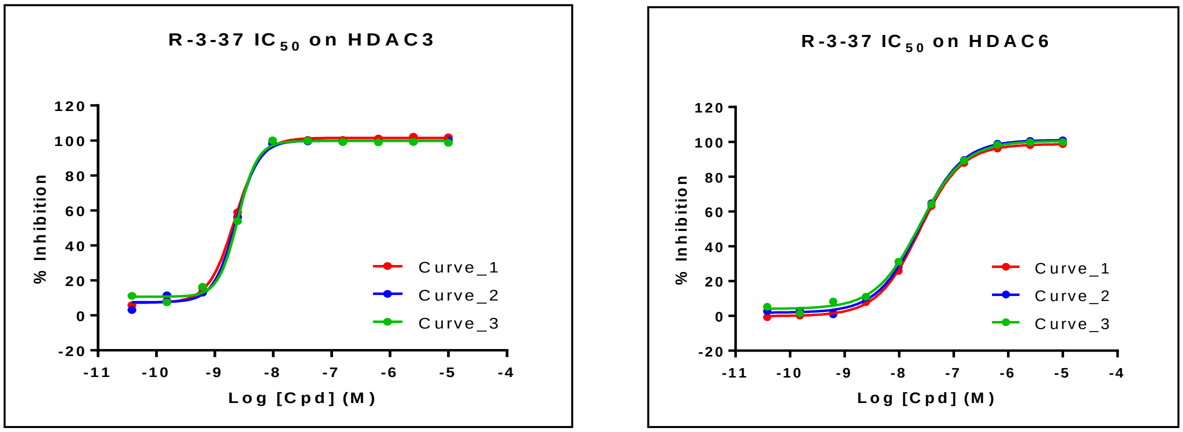

Figure S44**. The concentration-response curves of 15c in HDAC3 and HDAC6 assays (**Note: **15c** has been named as **R-3-37** in the figures.**)**

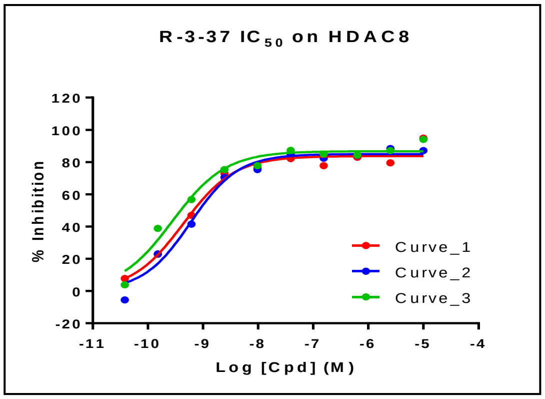

Figure S45**. The concentration-response curves of 15c in HDAC8 assays (**Note: **15c** has been named as **R-3-37** in the figure.**)**
